# Information Thermodynamics: From Physics to Neuroscience

**DOI:** 10.3390/e26090779

**Published:** 2024-09-11

**Authors:** Jan Karbowski

**Affiliations:** Institute of Applied Mathematics and Mechanics, Department of Mathematics, Informatics and Mechanics, University of Warsaw, 02-097 Warsaw, Poland; jkarbowski@mimuw.edu.pl

**Keywords:** information, non-equilibrium stochastic thermodynamics, computational neuroscience, learning, inference, neurons and synapses, plasticity

## Abstract

This paper provides a perspective on applying the concepts of information thermodynamics, developed recently in non-equilibrium statistical physics, to problems in theoretical neuroscience. Historically, information and energy in neuroscience have been treated separately, in contrast to physics approaches, where the relationship of entropy production with heat is a central idea. It is argued here that also in neural systems, information and energy can be considered within the same theoretical framework. Starting from basic ideas of thermodynamics and information theory on a classic Brownian particle, it is shown how noisy neural networks can infer its probabilistic motion. The decoding of the particle motion by neurons is performed with some accuracy, and it has some energy cost, and both can be determined using information thermodynamics. In a similar fashion, we also discuss how neural networks in the brain can learn the particle velocity and maintain that information in the weights of plastic synapses from a physical point of view. Generally, it is shown how the framework of stochastic and information thermodynamics can be used practically to study neural inference, learning, and information storing.

*“Earth, air, fire, and water in the end are all made of energy, but the different forms they take are determined by information. To do anything requires energy. To specify what is done requires information.”*— Seth Lloyd (2006) [1]

## 1. Introduction: Information Is Physical, So Is the Brain

Brain computations require a certain amount of energy [2,3,4,5,6,7], and the brain is one of the most metabolically expensive organs in the body [8]. Moreover, the brain energy cost (oxygen and glucose metabolic rates) scales linearly with the number of neurons [9] and sub-linearly with brain size [10]. Every transition in neural circuits, either on a microscopic or macroscopic scale, is associated with some energy dissipation [11,12,13,14,15,16,17,18]. Despite all this, a huge majority of neuronal models used in computational (or theoretical) neuroscience neglect completely the energetic aspect of brain functioning, as if neural information processing were for free and performed in some abstract “mathematical” hyperspace (e.g., [19,20,21,22,23]). One can argue that brain information processing is relatively cheap (only about 10–20 Watts for human brain [6,8]) in comparison to computations executed by artificial neural networks on semiconductor hardware (the supercomputer involved in the Blue Brain Project uses about $4\cdot 10^{5}$ Watts for a “realistic” simulation [24,25]). However, this relative brain energetic efficiency cannot be a justification for dismissing the metabolic constraints. In fact, handling information in real neural circuits is energetically demanding, as transmitting 1 bit of information through a chemical synapse requires about ∼$10^{5}$$k_{B}T$ of energy [4], and acquiring 1 bit by a synapse during synaptic learning needs a similar amount of ∼$5\cdot 10^{6}$$k_{B}T$ [18], where $k_{B}$ is the Boltzmann constant, and *T* is the brain temperature. Importantly, both these energy figures are much larger than the minimum set by the Landauer limit ($k_{B}T\ln2$; [26]). Most of the energy consumption in the mammalian brain goes for fast electric signaling, i.e., the generation of action potentials (neural activation) and synaptic transmission (each of them roughly $2\cdot 10^{8}$$k_{B}T$/min, for neuronal firing rates ∼5 Hz) [5,6,7], and for fast communication (spatial traveling of action potentials along axons) [27]. In contrast, slow chemical signaling associated with synaptic plasticity (related to learning and memory) requires much less energy, about 4–11% of the energy cost expanded on the synaptic transmission for low firing rates [13]. These substantial costs are likely the reason for observing sparse coding in brain networks, where only a small fraction of neurons and synapses are active at any instant of time [28,29]. All this suggests that energy is a strong constraint on neural information processing and storing, and consequently, not all sorts of computations, even theoretically possible, can be implemented by neural networks in the brain.

The first meaningful connection between physics and neuroscience was made a long time ago, in 1871, by James Maxwell in his book about heat [30]. In that book, he considered an “intelligent being” or “demon” that supposedly breaks the second law of thermodynamics by decreasing the entropy of the physical system. This thought experiment was a paradox that triggered a confusion regarding fundamental issues of thermodynamics and led to a huge amount of literature on this subject (for reviews, see [31,32]). The resolution of this paradox came with the realization that the concept of information also has to be included in the thermodynamic considerations, i.e., information has to be treated on equal footing with physical entropy and work [32].

This realization followed from a seminal observation made by Rolf Landauer that erasing information always leads to heat dissipation (erasure of 1 bit causes at least $k_{B}T\ln2$ of energy released into the environment [26]). In other words, information is physical, since its storing and processing requires physical hardware, and it has to comply with the laws of physics [33,34,35,36].

It seems that one of the main goals of neural networks of any brain is to accurately estimate the outside signals [21,37,38,39,40], which are relevant for the brain, using as little energy as possible [41,42,43]. Based on these estimates, the brain tries to predict the future dynamics of these signals and to plan action. The outside signals, or inputs coming to brain circuits, are mostly of a stochastic nature, and therefore, their estimation and prediction is additionally complicated and demanding. Given this, it is perhaps not surprising that the brain has to possess some internal, stable, representation of the outside world, which can be modified by learning. It is fair to say that despite many conceptual developments, we have only rudimentary knowledge (or feeling) of how this representation is created and works.

We can quantify the degree of correlation between outside dynamics and internal brain dynamics by mutual information, which is known from the Claude Shannon mathematical theory of communication [44]. This concept was brought to neuroscience by Horace Barlow [45] in the late 1950s. Much later, it was used by many neuroscientists, starting from Laughlin [46], Atick [37], and most notably by Bialek and colleagues [21,47,48]. These approaches aimed at the maximization of mutual information, initially ignoring energetic aspects. Levy and Baxter were likely the first to consider energetics of information encoding in neural networks [2,3]. However, even in these attempts, information and energy were treated as separate concepts that were not directly related to one another.

In contrast, stochastic thermodynamics provides a framework where information and energy are mutually related and can be considered and computed within a single formalism [36,49,50]. This is because on a micro-level, which includes molecular fluctuations, all relevant degrees of freedom have to be considered simultaneously. This work provides a perspective on a mutual connection between stochastic and information thermodynamics considered in physics and neural systems, which are intrinsically stochastic due to their small sizes and strong interactions with a fluctuating environment. This intrinsic stochasticity is a key ingredient of neurons and synapses that causes energy dissipation and influences information processing.

The paper is organized as follows. We start, in Section 2, with reviewing the fundamentals of stochastic dynamics and their relation to stochastic thermodynamics, with a simple pedagogic example of a Brownian particle moving in a gravitational field. This example is a basis and leitmotif for the next considerations, which link this stochastic mechanical system with the thermodynamics of information processing in neural networks. In Section 3, we introduce the relationship between entropy, information, and energy in general and in particular for the Brownian particle from Section 2. Next, in Section 4, we discuss information flow between two coupled subsystems, as a clear example where entropy production is directly related to information flow, and its relevance to the Maxwell demon. A neural network inferring the velocity of a Brownian particle (or a more general stochastic particle) is presented in Section 5 together with an associated energy cost. Synaptic plasticity and learning are discussed in Section 6 in the context of information gain and loss, using a stochastic version of the BCM model [51] together with its energy cost. It is shown here how the information loss after learning is related to the entropy production rate in synapses. Most of the calculations in Section 5 and Section 6 are novel; i.e., standard neural and synaptic models are analyzed in a new light. Finally, in Section 7, we briefly discuss a more general large-scale model of interacting plastic synapses during learning, using Glauber dynamics [52], in terms of information processing. We conclude with some general remarks about the relevance of information thermodynamics to neuroscience.

## 2. Stochastic Dynamics and Thermodynamics

### 2.1. Stochastic Dynamics

Small physical systems have internal degrees of freedom that are subject to fluctuations due to thermal noise (i.e., interactions with the environment or “heat bath”). These internal degrees of freedom can be described either by discrete or continuous time-dependent variables, such as position, velocity, activity, composition, etc. Let the index *z* denote an internal variable (or all relevant internal variables), describing the state of the system, and let p(z,t) denote the probability that the system is in this particular state at time *t*. Assuming that *z* follows a Markov process, one can describe the dynamics of the probability p(z,t) by a master equation [53,54]:

(1) $$\begin{array}{c} \dot{p}\left(z\right)=\sum_{z^{\prime }}\left(w_{zz^{\prime }}p\left(z^{\prime }\right)-w_{z^{\prime }z}p\left(z\right)\right), \end{array}$$

where $\dot{p}\left(z\right)$ denotes the temporal derivative of p(z), and $w_{zz^{\prime }}$ is the transition rate for jump from state $z^{\prime }$ to state *z*. Here, the variable *z* can be either discrete or continuous. In the latter case, one can expand Equation (1) to obtain the so-called Fokker–Planck equation (see below).

In the case with a single continuous internal variable z(t), we can write its stochastic dynamics as the so-called Langevin equation [53,54]:

(2) $$\begin{array}{c} \frac{1}{\mu }\dot{z}=F\left(z,t\right)+\sigma \eta \left(t\right), \end{array}$$

where F(z,t) is the deterministic generalized force acting on the system, which can depend on *z* and on time *t*, and μ is some parameter which is inversely proportional to the time scale of the dynamics. The parameter η(t) is the thermal noise acting on the variable *z* and thus can be described by a delta-correlated Gaussian random variable, such that 〈η(t)〉=0, and $\langle \eta \left(t\right)\eta \left(t^{\prime }\right)\rangle =\delta \left(t-t^{\prime }\right)$. The parameter σ characterizes the magnitude of the thermal noise. If *z* is velocity, then the two parameters, μ and σ, are not independent. In fact, they are mutually coupled by the temperature of the system *T* through the relation: $\sigma^{2}=2k_{B}T/\mu$ [53,54]. This relation is known as a fluctuation–dissipation theorem, which essentially means that in the presence of a heat bath (characterized by the temperature *T*), there is some balance between the level of fluctuations in the system (∼σ) and the time for which that system approaches equilibrium (∼$\mu^{-1}$). It should be noted that for neural systems, the thermal noise is not the most important source of noise, at least on the level of the whole neuron, and thus the temperature does not play a major part in the considerations of neural activation (see also below).

The dynamics of variable *z* can be described equivalently by the dynamics of probability density of the state variable *z* in terms of the Fokker–Planck equation as [53,54].

(3) $$\begin{array}{c} \frac{\partial P(z,t)}{\partial t}=-\frac{\partial J(z,t)}{\partial z}, \end{array}$$

with the probability current (or flux) J(z,t) given by

(4) $$\begin{array}{c} J\left(z,t\right)=\mu F\left(z,t\right)P\left(z,t\right)-\frac{1}{2}{\left(\mu \sigma \right)}^{2}\frac{\partial P(z,t)}{\partial z}, \end{array}$$

where P(z,t) is the probability density for the variable *z*.

In many circumstances, in physical systems, one thinks about *z* as a generalized position or velocity. In biological systems, *z* can be either some structural variable, concentration of some ions or molecules, or system activity. These are the most common “state variables”, although it should be noted that there are no restrictions about what physical observable a Langevin equation may or may not describe.

For concreteness, we take a specific example of Equation (2): a small particle of mass *m* moving in a gravitational field with some modulating time-dependent force $F_{0}\left(t\right)$ in the fluctuating environment with z(t) being the particle velocity v(t). This example will be our leitmotif for most of this paper, which is devoted to neural information processing and thermodynamics (Section 5 and Section 6). The Langevin equation of motion takes a familiar form:

(5) $$\begin{array}{c} m\dot{v}=-kv+F\left(t\right)+\sqrt{2mk}\sigma_{v}\eta \end{array}$$

where kv is the deterministic part of the resistance force of the environment with *k* being the parameter corresponding to the strength of the resistance and proportional to the size of the particle. The force F(t) is $F\left(t\right)=mg+F_{0}\left(t\right)$, with *g* being the gravitational acceleration, and $\sigma_{v}$ being the standard deviation (its steady-state value) of the particle velocity due to the thermal noise η acting on it (random hitting of air particles). When $F_{0}\left(t\right)=0$, the particle is falling freely with velocity-dependent friction and stochastic environmental fluctuations. In this case, at the steady state (t↦∞), we obtain the fluctuation–dissipation relation for our moving particle in the form $\sigma_{v}^{2}=k_{B}T/m$. This relation indicates that the fluctuations in the kinetic energy of the particle correspond to one degree of freedom associated with $k_{B}T/2$ (in 1D). It is instructive to have a sense of the magnitude of these fluctuations for real particles. For a particle with the size 0.1 mm and the mass of 1 μg (assuming the density 1 g/cm^3^), we obtain $\sigma_{v}=2$ μm/s, which is small and cannot be detected by a naked eye, but it can be observed with a microscope. For a comparison, for a hundred times greater particle with the size 1 cm and mass 1 g, we obtain $\sigma_{v}=0.002$ μm/s, which is extremely small.

We can write the Fokker–Planck equation for Equation (5), and easily solve it, yielding a Gaussian distribution $P_{v}$ for particle velocity [53]

(6) $$\begin{array}{c} P_{v}\left(v,t\right)=\frac{\exp\left(-{\left[v-\langle v\left(t\right)\rangle \right]}^{2}/2\sigma_{v}^{2}\left(t\right)\right)}{\sqrt{2\pi \sigma_{v}^{2}\left(t\right)}}, \end{array}$$

where 〈v(t)〉 is the average velocity, $\langle v\left(t\right)\rangle =\left[v\left(0\right)+\int_{0}^{t}dt^{\prime }e^{\gamma t^{\prime }}F\left(t^{\prime }\right)/m\right]e^{-\gamma t}$, with γ=k/m, and the time-dependent variance of velocity is $\sigma_{v}^{2}\left(t\right)=\sigma_{v}^{2}\left(1-e^{-2\gamma t}\right)=\langle v{\left(t\right)}^{2}\rangle -{\langle v\left(t\right)\rangle }^{2}$.

In the limit when the particle mass is very small, m↦0, we can neglect the term on the left in Equation (5) and use the fact that v=−dx/dt, with *x* being the height of the particle (velocity increases as height decreases). This corresponds to a standard overdamped approximation [55], and then Equation (5) transforms to

(7) $$\begin{array}{c} \dot{x}=-\frac{F(t)}{k}-\sqrt{2\gamma }\sigma_{x}\eta . \end{array}$$

This approximation is equivalent to saying that the particle velocity is in a quasi-stationary state, since its dynamic is governed by a fast time constant ∼m. In Equation (7), we used the rescaling $\sigma_{x}=\sigma_{v}/\gamma$, where $\sigma_{x}$ refers to the standard deviation of particle position *x*. We can also write the Fokker–Planck equation for the temporal evolution of the distribution of particle position $P_{x}\left(x,t\right)$ and easily solve it, obtaining

(8) $$\begin{array}{c} P_{x}\left(x,t\right)=\frac{\exp\left(-\frac{{\left[x-\langle x\left(t\right)\rangle \right]}^{2}}{4\sigma_{x}^{2}\gamma t}\right)}{\sqrt{4\pi \sigma_{x}^{2}\gamma t}}, \end{array}$$

where the average position $\langle x\left(t\right)\rangle =x\left(0\right)-\frac{1}{k}\int_{0}^{t}dt^{\prime }F\left(t^{\prime }\right)$. Additionally, the variance of particle position is $\langle x{\left(t\right)}^{2}\rangle -{\langle x\left(t\right)\rangle }^{2}=2\sigma_{x}^{2}\gamma t$, which indicates that it is growing proportionally with time, which is a characteristic of unrestricted Brownian motion. Also, in this limit, equivalent to the case γ≫1, we have a simple expression for the mean of particle velocity (as can be easily seen from Equation (7)), 〈v〉≈F(t)/k. Note that in contrast to the distribution for particle velocity (Equation (6)), which has a stationary solution, the distribution for particle position (Equation (8)) never assumes a stationary form.

### 2.2. Stochastic Thermodynamics

The first law of thermodynamics is essentially the rule for energy conservation. It turns out that Equation (2) can be used to derive the first law, as was realized by Sekimoto [56,57]. The idea is to treat the state variable in Equation (2) as generalized velocity and introduce an additional state variable *u* representing the generalized position, on which the generalized force also depends, i.e., F(z,u,t), with z=du/dt. Next, we decompose the force F(z,u,t) as $F\left(z,u,t\right)=-\partial V(u,t)/\partial u+f_{nc}\left(z\right)$, where V(u,t) is the generalized potential (dependent on *u* and *t*), and $f_{nc}\left(z\right)$ is the generalized nonconservative force (dependent on velocity *z*). After the multiplication of both sides of Equation (2) by *z* and rearrangement, we obtain the conservation of generalized “mechanical energy” in the following form:

(9) $$\begin{array}{c} \frac{d}{dt}\left(\frac{1}{2}\mu^{-1}z^{2}+V\left(u,t\right)\right)=\frac{\partial V(u,t)}{\partial t}+zf_{nc}\left(z\right)+\sigma z\eta \left(t\right), \end{array}$$

where we used the differentiation rule $dV\left(u,t\right)/dt=\frac{\partial V(u,t)}{\partial t}+\frac{\partial V(u,t)}{\partial u}\dot{u}$. Note that the left-hand side of Equation (9) is the temporal rate of mechanical energy, represented by $\frac{1}{2}\mu^{-1}z^{2}+V\left(u,t\right)$, which is the sum of “kinetic energy” (with $\mu^{-1}$ representing the generalized mass) and generalized potential V(u,t). Equation (9) implies that mechanical energy is lost (or gained) in three different ways: by temporal changes in the external potential *V*, by the action of nonconservative force $f_{nc}$, and by the noise (∼η). The last two factors constitute the heat dissipated to the environment.

In the case of our Brownian particle in the gravitational field, we find the law of energy conservation as

(10) $$\begin{array}{c} \frac{dE_{mech}}{dt}=-kv^{2}+vF_{0}\left(t\right)+\sqrt{2km}\sigma_{v}v\eta , \end{array}$$

where $E_{mech}$ is the mechanical energy of the particle, $E_{mech}=\frac{1}{2}mv^{2}+mgx$. Averaging this equation over the distribution of velocities, Equation (6), yields the mean balance of energy loss and gain:

(11) $$\begin{array}{c} \frac{d\langle E_{mech}\rangle }{dt}=-k\langle v^{2}\rangle +\langle v\rangle F_{0}\left(t\right)+k\sigma_{v}^{2}, \end{array}$$

where we used the Novikov theorem [58] for determining the average $\langle v\eta \rangle =\sigma_{v}\sqrt{k/(2m)}$. According to our expectations, the mean mechanical energy is lost due to friction (the term −$k\langle v^{2}\rangle$), and $\langle E_{mech}\rangle$ can be either decreased or increased by the driving force depending on its sign. But interestingly, $\langle E_{mech}\rangle$ is always increased by the presence of thermal fluctuations (the term $k\sigma_{v}^{2}$).

Equation (11) in the limit m↦0, equivalent to γ≫1, and corresponding to the unrestricted Brownian motion [Equations (7) and (8)], takes a simple form

(12) $$\begin{array}{c} \frac{d\langle E_{mech}\rangle }{dt}\approx -k{\langle v\rangle }^{2}+F_{0}\left(t\right)\langle v\rangle \\ \approx -\frac{mg}{k}\left[mg+F_{0}\left(t\right)\right]. \end{array}$$

Thus, the rate of mean mechanical energy is negative unless the driving force is negative (breaking from outside) and sufficiently strong. This means that opposing the gravitational force can save the mean mechanical energy or even increase it. We will come back also to the mechanical energy later in the context of entropy production and flux.

## 3. Entropy, Information, and the Second Law of Thermodynamics

### 3.1. Entropy, Kullback–Leibler Divergence, and Information

For the system with probability p(z,t) described by Equation (1) one can define Shannon entropy $S_{z}\left(t\right)$ as [44,59]

(13) $$\begin{array}{c} S_{z}\left(t\right)=-\sum_{z}p\left(z,t\right)\ln p\left(z,t\right), \end{array}$$

which is the measure of an average uncertainty about the state of the system or the value of the stochastic variable *z*. The larger the entropy, the less is known about the actual state of the system. The concept of entropy is central in thermodynamics [31,32,49], in information theory [59], and in the science of complexity [60].

It is worth noting that Shannon entropy is not the only way to define entropy. There are other definitions of entropy, such as Renyi entropy [61,62] and Tsallis entropy [63], which are also used in statistical physics and information theory [64,65,66]. Shannon entropy in Equation (13) is a special case of these more general entropies.

For two different probability distributions describing the same physical system, i.e., p(z) and q(z), one can define a statistical distance between them (in fact, it is a pseudo-distance in probability space) called Kullback–Leibler (KL) divergence [59,67]

(14) $$\begin{array}{c} D_{KL}\left(p||q\right)=\sum_{z}p\left(z\right)\ln\frac{p(z)}{q(z)}. \end{array}$$

KL divergence is also called the relative entropy, and it is always non-negative and quantifies the difference between the distributions p(z) and q(z). Therefore, $D_{KL}\left(p||q\right)$ can be also thought as an information gain by observing the p(z) distribution in relation to the baseline distribution q(z). The larger the KL divergence, the more distinct the two distributions are. $D_{KL}$ has many applications in statistical physics and information theory [59,68]. We will use it in the following sections for synaptic information gain and loss.

As for the entropy, one can define also other statistical divergences, such as Renyi and Tsallis divergences [61,63]. $D_{KL}$ is a special case of these more general divergences. There exist numerous inequalities relating various types of statistical divergences [62,69] and inequalities relating the rates of these divergences to stochastic thermodynamics [70].

In the case of two coupled systems described by variables *x* and *y*, one can write z=(x,y) and define the joint probability $p_{xy}$ as well as marginal probability distributions $p_{x}$ and $p_{y}$ for each subsystem separately. This allows us to introduce the measure of mutual dependency between the two subsystems, $\ln\frac{p_{xy}}{p_{x}p_{y}}$, which is zero if *x* and *y* are independent and nonzero otherwise. The average of this quantity over all realizations of x,y is called the mutual information $I_{xy}$ between *x* and *y* [59]

(15) $$\begin{array}{c} I_{xy}=\sum_{x,y}p_{xy}\ln\frac{p_{xy}}{p_{x}p_{y}}\\ \equiv D_{KL}(p_{xy}\left|\right|p_{x}p_{y}). \end{array}$$

Thus, the mutual information is the KL divergence between the joint probability $p_{xy}$ and the product of marginal probabilities $p_{x},p_{y}$. The definition in Equation (15) ensures that mutual information is always non-negative, and the stronger the dependence between *x* and *y*, the larger $I_{xy}$. This is in contrast to entropy, which can be negative for continuous probability distributions (when summation is replaced by integration).

From Equation (15), it follows that the mutual information can be also represented in terms of entropies [59]:

(16) $$\begin{array}{c} I_{xy}=S_{x}-S_{x|y}=S_{y}-S_{y|x}, \end{array}$$

where $S_{x|y}$ is the conditional entropy defined as $S_{x|y}=-\sum_{x,y}p_{xy}\ln p\left(x|y\right)$, with p(x|y) denoting the conditional probability, $p\left(x|y\right)=p_{xy}/p_{y}$, and similarly for for reverse conditional entropy $S_{y|x}$ and conditional probability p(y|x).

In recent years, information theory in general, and mutual information in particular, were applied to stochastic processes in different settings. For example, information theory was used to derive thermodynamic uncertainty relations [71]. Mutual information can be helpful in mapping the input trajectory to the output trajectory, which is relevant for biochemical networks [72]. Additionally, mutual information can be used to discriminate between internal information in the system and the information coming from external sources [73], which may have some relevance in neuroscience. In the latter context, mutual information was shown to be maximized for critical brain states with power law distributions of neural activity [74,75]. In a broader biological context, it has been argued that evolution acts to optimize the gathering and representation of information across many spatial scales [48].

### 3.2. Entropy Production and Flow, and the Second Law

The temporal derivative of the entropy from Equation (13) can be decomposed into two contributions [76,77,78]:

(17) $$\begin{array}{c} \frac{dS}{dt}={\dot{S}}_{pr}-{\dot{S}}_{fl}, \end{array}$$

where ${\dot{S}}_{pr}$ is the entropy production rate given by

(18) $$\begin{array}{c} {\dot{S}}_{pr}=\frac{1}{2}\sum_{z,z^{\prime }}\left(w_{zz^{\prime }}p_{z^{\prime }}-w_{z^{\prime }z}p_{z}\right)\ln\frac{w_{zz^{\prime }}p_{z^{\prime }}}{w_{z^{\prime }z}p_{z}}, \end{array}$$

and ${\dot{S}}_{fl}$ is the entropy flow rate given by

(19) $$\begin{array}{c} {\dot{S}}_{fl}=\frac{1}{2}\sum_{z,z^{\prime }}\left(w_{zz^{\prime }}p_{z^{\prime }}-w_{z^{\prime }z}p_{z}\right)\ln\frac{w_{zz^{\prime }}}{w_{z^{\prime }z}}. \end{array}$$

The thermodynamic interpretation of ${\dot{S}}_{fl}$ is that it is proportional to the heat ΔQ exchanged with the surrounding medium, i.e., $\Delta Q=k_{B}T{\dot{S}}_{fl}\Delta t$, in the short time interval Δt. Moreover, the entropy flow can be of either sign, which reflects the fact that the system can either gain energy from the environment (${\dot{S}}_{fl}<0$) or dissipate energy into the environment (${\dot{S}}_{fl}>0$).

The entropy production rate, on the other hand, is always non-negative, which follows from the fact that the two factors on the right in Equation (18) have the same signs, which are either both positive or negative. Alternatively, the non-negativity of ${\dot{S}}_{pr}$ and its lower bound can be determined from a well-known inequality, $\ln\left(1+x\right)\geq \frac{x}{1+x}$, which is valid for all x>−1. Applying this to Equation (18) leads to

(20) $$\begin{array}{c} {\dot{S}}_{pr}\geq \frac{1}{2}\sum_{z,z^{\prime }}\frac{{\left(w_{zz^{\prime }}p_{z^{\prime }}-w_{z^{\prime }z}p_{z}\right)}^{2}}{w_{zz^{\prime }}p_{z^{\prime }}}\geq 0. \end{array}$$

The fact that ${\dot{S}}_{pr}\geq 0$ has a tremendous consequence on the behavior of stochastic objects in the form of the second law of thermodynamics. In a nutshell, the second law says that the entropy of the isolated physical system (for which ${\dot{S}}_{fl}=0$) never decreases, i.e., $dS/dt={\dot{S}}_{pr}\geq 0$, which means that disorder of the isolated system tends to increase over time.

Equations (18) and (19) apply to the general case described by the master Equation (1); however, it is also possible to define ${\dot{S}}_{pr}$ and ${\dot{S}}_{fl}$ for continuous stochastic variables described by the Fokker–Planck Equations (3) and (4). In the latter case, we have [79]

(21) $$\begin{array}{c} {\dot{S}}_{pr}=\frac{2}{{\left(\mu \sigma \right)}^{2}}\int dz\;\frac{J{\left(z,t\right)}^{2}}{P(z,t)}\geq 0, \end{array}$$

and

(22) $$\begin{array}{c} {\dot{S}}_{fl}=\frac{2}{\mu \sigma^{2}}\int dz\;J\left(z,t\right)F\left(z,t\right). \end{array}$$

For the system at steady state, i.e., for $\dot{p}\left(z,t\right)=0$, its entropy is constant with dS/dt=0, which implies ${\dot{S}}_{pr}={\dot{S}}_{fl}$. This equality can happen in two cases. In the first, the probability flux J(z,t)=0 for continuous variables, and $w_{zz^{\prime }}p_{z^{\prime }}-w_{z^{\prime }z}p_{z}=0$ for discrete variables. This situation describes the so-called detailed balance (where all local probability fluxes balance each other), which corresponds to the thermodynamic equilibrium with the environment. In the second case, one can have nonzero probability flux, J(z,t)≠0, and broken detailed balance $w_{zz^{\prime }}p_{z^{\prime }}-w_{z^{\prime }z}p_{z}\neq 0$. This situation takes place in the so-called driven systems by outside factors that provide energy and materials for maintaining the steady state out of equilibrium with the environment. Such a steady state is called a non-equilibrium steady state (NESS) [49,50]. All biological systems are out of equilibrium [11,33,80], and many biological processes operate in a non-equilibrium steady state [39,49], including neural systems [13,14].

Since at steady state ${\dot{S}}_{pr}={\dot{S}}_{fl}$, one can say roughly that for any conditions, the entropy production rate is proportional to the amount of dissipated energy to the environment. Thus, it is useful to think about ${\dot{S}}_{pr}$ as a measure of the energy cost of performing a non-trivial function that requires non-equilibrium conditions.

### 3.3. Entropy Production and Flow for the Brownian Particle

Our Brownian particle falling in the gravitational field represented by Equations (5)–(8) has entropy (Equation (13)) corresponding to its position distribution $P_{x}\left(x,t\right)$ given by [59]

(23) $$\begin{array}{c} S_{x}\left(t\right)=\frac{1}{2}\ln\left(4\pi e\sigma_{x}^{2}\gamma t\right), \end{array}$$

which grows logarithmically with time. This means that the uncertainty about the particle position increases weakly with time. However, the entropy rate, $dS_{x}/dt$, decreases with time as

(24) $$\begin{array}{c} \frac{dS_{x}}{dt}=\frac{1}{2t}. \end{array}$$

The entropy production rate for particle position (the main “state variable”) can be found from Equation (21). For this, we need the probability current J(x,t) (Equation (4)) for our particle position, which is

(25) $$\begin{array}{c} J\left(x,t\right)=\left(-\frac{F(t)}{k}+\frac{\left[x-\langle x\rangle \right]}{2t}\right)P_{x}\left(x,t\right). \end{array}$$

This allows us to find the entropy production rate ${\dot{S}}_{pr,x}$ in the form

(26) $$\begin{array}{c} {\dot{S}}_{pr,x}=\frac{1}{2t}+\frac{{\left[mg+F_{0}\left(t\right)\right]}^{2}}{\gamma k^{2}\sigma_{x}^{2}}. \end{array}$$

Note that when there is no driving force ($F_{0}=0$), the entropy production rate decreases all the time to its asymptotic value ${\left(mg\right)}^{2}/\left(\gamma k^{2}\sigma_{x}^{2}\right)=m^{3}g^{2}/\left(k^{3}\sigma_{x}^{2}\right)$.

The entropy flux rate can be quickly found from Equations (24) and (26), using the definition (17). The result is

(27) $$\begin{array}{c} {\dot{S}}_{fl,x}=\frac{{\left[mg+F_{0}\left(t\right)\right]}^{2}}{\gamma k^{2}\sigma_{x}^{2}}\\ \approx \frac{k{\langle v\rangle }^{2}}{k_{B}T}, \end{array}$$

where the second approximate equality comes from using the fluctuation–dissipation theorem and the approximate equality for the average particle velocity $\langle v\rangle \approx [mg+F_{0}\left(t\right)]/k$ (see Equation (7)). Thus, in this case, the entropy flux is always positive, suggesting energy dissipation to the environment.

The relationship between the mechanical energy loss and the entropy flux is (from Equations (12) and (27))

(28) $$\begin{array}{c} \frac{d\langle E_{mech}\rangle }{dt}\approx -k_{B}T{\dot{S}}_{fl,x}+F_{0}\left(t\right)\langle v\rangle . \end{array}$$

This equation is the manifestation of the first law of thermodynamics or equivalently the law of energy conservation. It implies that our (mechanical) system changes its energy $E_{mech}$ by dissipating heat to the environment ($k_{B}T{\dot{S}}_{fl,x}$) and by mechanical work performed on the particle by the external force $F_{0}$. Equation (28) also suggests that the rate of the mean mechanical energy of the Brownian particle is related to the entropy flux rate for its position, but they are not the same. The energy lost $d\langle E_{mech}\rangle /dt$ and ${\dot{S}}_{fl,x}$ are directly proportional only if $F_{0}=0$. To conclude, the entropy flux rate is a measure of dissipated energy (heat), but it does not account for all the lost or gained energy of the system.

## 4. Information Flow between Two Subsystems and the Maxwell Demon

In this section, we follow closely the main ideas presented in Ref. [81]. Consider two coupled subsystems *X* and *Y* with dynamics of the joint probability $p_{xy}$ described by the following master equation

(29) $$\begin{array}{c} {\dot{p}}_{xy}=\sum_{x^{\prime }}\left(w_{xx^{\prime }}^{y}p_{x^{\prime }y}-w_{x^{\prime }x}^{y}p_{xy}\right)\\ +\sum_{y^{\prime }}\left(w_{yy^{\prime }}^{x}p_{xy^{\prime }}-w_{y^{\prime }y}^{x}p_{xy}\right), \end{array}$$

where $w_{xx^{\prime }}^{y}$ is the transition rate in the subsystem *X* from state $x^{\prime }$ to state *x*, which depends on the actual state *y* of the second subsystem *Y* (and similarly for $w_{yy^{\prime }}^{x}$). The form of the master equation in Equation (29) has a bipartite structure, in which simultaneous jumps in the two subsystems are neglected as much less likely than single jumps.

For this system, we can define the rate of mutual information $dI_{xy}/dt$ as [81,82]

(30) $$\begin{array}{c} \frac{dI_{xy}}{dt}={\dot{I}}_{x}+{\dot{I}}_{y}, \end{array}$$

where ${\dot{I}}_{x}=\left[I_{x_{t+dt},y_{t}}-I_{x_{t},y_{t}}\right]/dt$, and ${\dot{I}}_{y}=\left[I_{x_{t},y_{t+dt}}-I_{x_{t},y_{t}}\right]/dt$, with dt↦0. The explicit expressions for ${\dot{I}}_{x}$ and ${\dot{I}}_{y}$ are given by [81]

(31) $$\begin{array}{c} {\dot{I}}_{x}=\sum_{x>x^{\prime },y}\left(w_{xx^{\prime }}^{y}p_{x^{\prime }y}-w_{x^{\prime }x}^{y}p_{xy}\right)\ln\frac{p(y|x)}{p(y|x^{\prime })}, \end{array}$$

and

(32) $$\begin{array}{c} {\dot{I}}_{y}=\sum_{y>y^{\prime },x}\left(w_{yy^{\prime }}^{x}p_{xy^{\prime }}-w_{y^{\prime }y}^{x}p_{xy}\right)\ln\frac{p(x|y)}{p(x|y^{\prime })}. \end{array}$$

The essence of the decomposition in Equation (30) is that it splits the total rate of mutual information into two flows of information. The first flow, ${\dot{I}}_{x}$, relates to the change in mutual information between the two subsystems that is only due to the dynamics of *X*. The second flow, ${\dot{I}}_{y}$, is analogous and relates to *Y*. When ${\dot{I}}_{x}>0$, then information is created in the subsystem *X* as it monitors the *Y* subsystem.

In the same manner, we can split the rate of entropy of the joint system (X,Y), i.e., $dS_{xy}/dt$, as well as the joint entropy production rate ${\dot{S}}_{pr,xy}$ and the joint entropy flux rate ${\dot{S}}_{fl,xy}$. We have

(33) $$\begin{array}{c} \frac{dS_{xy}}{dt}={\dot{S}}_{x}+{\dot{S}}_{y}, \end{array}$$

where $S_{xy}=-\sum_{x,y}p_{xy}\ln p_{xy}$, and the rates of entropy in each subsystem ${\dot{S}}_{x}$ and ${\dot{S}}_{y}$ are given by

(34) $$\begin{array}{c} {\dot{S}}_{x}=-\sum_{y}\sum_{x>x^{\prime }}\left(w_{xx^{\prime }}^{y}p_{x^{\prime }y}-w_{x^{\prime }x}^{y}p_{xy}\right)\ln p_{xy}, \end{array}$$

and

(35) $$\begin{array}{c} {\dot{S}}_{y}=-\sum_{x}\sum_{y>y^{\prime }}\left(w_{yy^{\prime }}^{x}p_{xy^{\prime }}-w_{y^{\prime }y}^{x}p_{xy}\right)\ln p_{xy}. \end{array}$$

Note that in the particular case of two independent subsystems, we have $w_{xx^{\prime }}^{y}\mapsto w_{xx^{\prime }}$ ($w_{yy^{\prime }}^{x}\mapsto w_{yy^{\prime }}$), and the subsystems entropy rates ${\dot{S}}_{x}$ and ${\dot{S}}_{y}$ reduce to ${\dot{S}}_{x}=-\sum_{x}{\dot{p}}_{x}\ln p_{x}$ and ${\dot{S}}_{y}=-\sum_{y}{\dot{p}}_{y}\ln p_{y}$, i.e., in agreement with the expectations.

Similarly, the joint entropy production ${\dot{S}}_{pr,xy}$ and entropy flux ${\dot{S}}_{fl,xy}$ can be decomposed as

(36) $$\begin{array}{c} {\dot{S}}_{pr,xy}={\dot{S}}_{pr,x}+{\dot{S}}_{pr,y}, \end{array}$$

where

(37) $$\begin{array}{c} {\dot{S}}_{pr,x}=\sum_{x>x^{\prime },y}\left(w_{xx^{\prime }}^{y}p_{x^{\prime }y}-w_{x^{\prime }x}^{y}p_{xy}\right)\ln\frac{w_{xx^{\prime }}^{y}p_{x^{\prime }y}}{w_{x^{\prime }x}^{y}p_{xy}},\\ {\dot{S}}_{pr,y}=\sum_{y>y^{\prime },x}\left(w_{yy^{\prime }}^{x}p_{xy^{\prime }}-w_{y^{\prime }y}^{x}p_{xy}\right)\ln\frac{w_{yy^{\prime }}^{x}p_{xy^{\prime }}}{w_{y^{\prime }y}^{x}p_{xy}}, \end{array}$$

and for the entropy flux

(38) $$\begin{array}{c} {\dot{S}}_{fl,xy}={\dot{S}}_{fl,x}+{\dot{S}}_{fl,y}, \end{array}$$

where

(39) $$\begin{array}{c} {\dot{S}}_{fl,x}=\sum_{x>x^{\prime },y}\left(w_{xx^{\prime }}^{y}p_{x^{\prime }y}-w_{x^{\prime }x}^{y}p_{xy}\right)\ln\frac{w_{xx^{\prime }}^{y}}{w_{x^{\prime }x}^{y}},\\ {\dot{S}}_{fl,y}=\sum_{y>y^{\prime },x}\left(w_{yy^{\prime }}^{x}p_{xy^{\prime }}-w_{y^{\prime }y}^{x}p_{xy}\right)\ln\frac{w_{yy^{\prime }}^{x}}{w_{y^{\prime }y}^{x}}. \end{array}$$

The terms ${\dot{S}}_{pr,x},{\dot{S}}_{pr,y}$ can be interpreted as local entropy production rates, while ${\dot{S}}_{fl,x},{\dot{S}}_{fl,y}$ are local entropy fluxes. As before, ${\dot{S}}_{pr,x}$ and ${\dot{S}}_{pr,y}$ are both non-negative, which means that the second law is valid also in each of the subsystems.

The interesting thing coming from all these equations is that local entropy productions ${\dot{S}}_{pr,x}$, ${\dot{S}}_{pr,y}$ are related to information flows ${\dot{I}}_{x}$ and ${\dot{I}}_{y}$ as [81]

(40) $$\begin{array}{c} {\dot{S}}_{pr,x}={\dot{S}}_{x}+{\dot{S}}_{fl,x}-{\dot{I}}_{x},\\ {\dot{S}}_{pr,y}={\dot{S}}_{y}+{\dot{S}}_{fl,y}-{\dot{I}}_{y}. \end{array}$$

These equations imply that local entropy balance involves both energy dissipation (${\dot{S}}_{fl,x},{\dot{S}}_{fl,y}$) and the flow of information (${\dot{I}}_{x},{\dot{I}}_{y}$). Consequently, energy and information are mutually coupled, and one influences the other. Equation (40) provides an important link between information processing and its energy cost.

How do the results represented by Equation (40) relate to the Maxwell demon? Although the quantity ${\dot{S}}_{pr,x}$ always satisfies ${\dot{S}}_{pr,x}\geq 0$, the sum ${\dot{S}}_{x}+{\dot{S}}_{fl,x}$ can be negative if the information flow ${\dot{I}}_{x}<0$. Thus, from a local point of view of the subsystem *X*, its visible “entropy production” (i.e., ${\dot{S}}_{x}+{\dot{S}}_{fl,x}$) can be negative if the presence of the *Y* subsystem is neglected. This seems like a violation of the second law (requiring positive entropy production rate), and it is closely related to the Maxwell demon thought experiment. Obviously, the inclusion of the information flow term ${\dot{I}}_{x}$ in the local entropy production solves the paradox.

## 5. Neural Inference

In this section, we consider a simple model of how neurons estimate an external signal. We will discuss this model in terms of information processing as well as thermodynamics.

Neurons in the visual cortex selectively respond to different velocities of a moving stimulus [21,83]. Generally, each neuron has a preferred velocity to which it responds in the form of an elevated firing rate (it is called a tuning curve, see, e.g., [19,21]). Thus, a single neuron is unable to estimate (decode) the velocity of the moving stimulus, because it reacts only to a very small range of velocities. However, a large population of neurons can do it, although with only some accuracy. Below, we consider how such a decoding can take place. In the example below, which is mostly a “thought experiment”, the moving stimulus should be a particle with a substantial size and velocity to be detectable by visual neurons. Typical Brownian particles are too small and too slow to be directly observable by the mammalian visual system. To make them observable, a magnifying instrument such as a microscope is needed. Thus, one can think about the moving stimulus below as a magnified Brownian particle from Section 2, or alternatively, as a macroscopic object moving stochastically, e.g., due to strong stochastic force $F_{0}$ not related to thermal fluctuations of the environment. The analysis below is independent of either choice.

The model we use is a stochastic version of the deterministic model called a linear recurrent network for interacting neurons (see Equation (7.17) in [19]). In this model, the activity or firing rate $r_{i}$ (number of action potentials or spikes per time unit) of a single neuron labeled as *i* in the visual cortex can be represented as

(41) $$\begin{array}{c} {\dot{r}}_{i}=-\frac{\left(r_{i}-c_{i}\left(v\right)\right)}{\tau_{n0}}+\frac{1}{N}\sum_{j}w_{ij}r_{j}+\sqrt{\frac{2\sigma_{r0}^{2}}{\tau_{n0}}}\eta_{i}\left(t\right), \end{array}$$

where $w_{ij}$ is the synaptic weight (or strength) characterizing the magnitude of synaptic transmission coming from neuron *j*, and i=1,2,…,N, with *N* number of neurons in the network (here, $w_{ij}$ are in units of inverse of time). Since the majority of synapses in the cortex of mammals is excitatory (about 80–90%; [84,85]), the weights $w_{ij}$ are assumed to be positive, which implies that the steady-state average values of $r_{i}$ are all positive. The parameter $\tau_{n0}$ is the time constant of the single neuron dynamics related to changes in its firing rate, and $\sigma_{r0}$ represents the standard deviations of the Gaussian noise $\eta_{i}$ related to firing rate fluctuations. The function $c_{i}\left(v\right)$ is the sensory input coming to neuron *i*, which is discussed below. The activity of the neuron *i* is a compromise between this sensory input and the synaptic contributions coming from other neurons in the network. It should be also clearly stated that the noise term in Equation (41) is not of thermal origin. Microscopic thermal fluctuations present in synapses and different ion channels have only a marginal influence on neural activity, since their numbers for a typical cortical neuron are very large (small variance), although there are some exceptions (see [86]). More important are the fluctuations caused by an unreliable sensory signal and unpredictable synaptic transmission (probabilistic neurotransmitter release), the latter being caused by the low numbers of signaling molecules involved [87].

Before we go further, let us talk about the range of validity of Equation (41). First, both the linear term associated with synaptic interactions and the additive noise can occasionally make the firing rate $r_{i}$ negative, which is obviously wrong (even if all synaptic weights are positive). However, this can happen only transiently, especially in the limit of weak noise. Moreover, the steady-state average values of firing rates are always positive, since on average, the term $\sum_{j}w_{ij}r_{j}$ is positive. This means that the linear approximation is a “reasonable” approximation, and we use it primarily because such a linear model can be analytically analyzed, revealing some generic features. Second, the time constant $\tau_{n0}$ in Equation (41) cannot be too small. It must be significantly larger than a time constant related to synaptic transmission (5 ms and 120 ms, related to AMPA and NMDA synaptic receptors), such that synaptic currents assume quasi-stationary values [19]. In what follows, i.e., the analysis of the dynamics and information aspects of this model, is a novel calculation.

The sensory input $c_{i}\left(v\right)$ received by neuron *i* is in these particular settings also called the tuning curve for neuron *i*. It can be approximated by a Gaussian as (see Equation (3.28) in [19])

(42) $$\begin{array}{c} c_{i}\left(v\right)=r_{m}\exp\left[-\frac{{\left(v-u_{i}\right)}^{2}}{2\epsilon^{2}}\right], \end{array}$$

where $r_{m}$ is the maximal firing rate in response to the visual stimulus (the same for all neurons in the network), $u_{i}$ is the preferred velocity for the neuron *i*, and ϵ characterizes the maximal deviation from the preferred velocity for which neurons are still (weakly) activated. We take ϵ to be small, i.e., typically $\epsilon /u_{i}\ll 1$. Note that for $v=u_{i}$, we have $c_{i}\left(v\right)=r_{m}$, while for $v=u_{i}\pm 2\epsilon$, we have $c_{i}\left(v\right)=0.14r_{m}$. Equation (41) indicates that the neuron adjusts dynamically to the changes in the stimulus (in its sensitivity range represented by $c_{i}\left(v\right)$) and in the synaptic input coming from other neurons.

Since the decoding of stimulus velocity is a collective process, we define a population average of all neural activities, denoted as $\bar{r}$, and defined as $\bar{r}=\left(1/N\right)\sum_{i=1}^{N}r_{i}$. Consequently, the dynamic of the population average neural activity $\bar{r}$ can be represented as

(43) $$\begin{array}{c} \dot{\bar{r}}=-\frac{\left[\bar{r}-\kappa \left(\bar{w}\right)\bar{c}\left(v\right)\right]}{\tau_{n}}+\sqrt{\frac{2\sigma_{r}^{2}}{N\tau_{n}}}\bar{\eta }\left(t\right), \end{array}$$

where we made a mean-field type approximation $\left(1/N^{2}\right)\sum_{i,j}w_{ij}r_{j}\approx \bar{w}\bar{r}$, where $\bar{w}$ is the population average synaptic weight, i.e., $\bar{w}=\left(1/N^{2}\right)\sum_{i}\sum_{j}w_{ij}$. We assume that $\bar{w}>0$, which follows from the fact that the majority of synapses are excitatory [84,85]. The term $\bar{c}\left(v\right)$ is the population average tuning curve, $\bar{c}\left(v\right)=\left(1/N\right)\sum_{i=1}^{N}c_{i}\left(v\right)$, which is given by (see Appendix A)

(44) $$\begin{array}{c} \bar{c}\left(v\right)\approx \frac{r_{m}\epsilon }{\alpha }\exp\left(-\frac{v^{2}}{2\alpha^{2}}\right)\\ \approx \frac{r_{m}\epsilon }{\alpha }\left[1-\frac{v^{2}}{2\alpha^{2}}+O\left(\alpha^{-4}\right)\right], \end{array}$$

where α is the velocity range to which neurons respond. The approximate equality in Equation (44) follows from the fact that α is generally large, i.e., α≫1, and we will use that approximation in the calculations below. The parameter $\kappa (\bar{w})$ is the network enhancement factor given by

(45) $$\begin{array}{c} \kappa \left(\bar{w}\right)=\frac{1}{\left(1-\bar{w}\tau_{n0}\right)}, \end{array}$$

since for $\bar{w}\mapsto \tau_{n0}^{-1}$, the parameter $\kappa (\bar{w})\mapsto \infty$ (obviously, we must assume that $\bar{w}<\tau_{n0}^{-1}$). The parameter $\tau_{n}$ is the effective time constant of the neural population dynamics $\tau_{n}=\kappa \left(\bar{w}\right)\tau_{n0}$, and $\bar{\eta }$ is the population-averaged noise, i.e., $\bar{\eta }=\left(1/\sqrt{N}\right)\sum_{i=1}^{N}\eta_{i}$ with zero mean and unit variance, with $\sigma_{r}$ being the effective standard deviation of the noise in the network, i.e., $\sigma_{r}=\sqrt{\kappa (\bar{w})}\sigma_{r0}$. Note that the main effect of the network interactions on population dynamics, as compared to the single neuron dynamics, is to significantly enhance the tuning curve, the time constant, and the standard deviation.

Equation (43) corresponds to the time-dependent distribution of mean neural activity conditioned on stimulus velocity *v*, $\rho (\bar{r}|v,t)$, which is in the form [53]

(46) $$\begin{array}{c} \rho \left(\bar{r}|v,t\right)=\frac{\exp\left(-N{\left[\bar{r}-{\langle \bar{r}\left(v,t\right)\rangle }_{\rho (\bar{r}|v)}\right]}^{2}/2\sigma_{r}^{2}\left(t\right)\right)}{\sqrt{2\pi \sigma_{r}^{2}\left(t\right)/N}}, \end{array}$$

where $\sigma_{r}^{2}\left(t\right)=\sigma_{r}^{2}\left(1-e^{-2t/\tau_{n}}\right)$, and ${\langle \bar{r}\left(v,t\right)\rangle }_{\rho (\bar{r}|v)}$ is the stochastic average of the population mean of neural activity over the conditional distribution $\rho (\bar{r}|v,t)$, i.e., ${\langle \bar{r}\left(v,t\right)\rangle }_{\rho (\bar{r}|v)}=\int \;d\bar{r}\rho \left(\bar{r}|v,t\right)\bar{r}$. The latter can be found quickly by averaging Equation (43) over noise and then by finding its time-dependent solution. The result is

(47) $$\begin{array}{c} {\langle \bar{r}\left(v,t\right)\rangle }_{\rho (\bar{r}|v)}=\bar{r}\left(0\right)e^{-t/\tau_{n}}+\frac{\epsilon \kappa r_{m}}{\alpha }\left(1-e^{-t/\tau_{n}}\right)\\ -\frac{\epsilon \kappa r_{m}}{2\tau_{n}\alpha^{3}}e^{-t/\tau_{n}}\int_{0}^{t}\;dt^{\prime }\;e^{t^{\prime }/\tau_{n}}v^{2}\left(t^{\prime }\right)+O\left(\alpha^{-5}\right), \end{array}$$

where $\bar{r}\left(0\right)$ is the initial mean neural activity. This equation indicates that the outside stimulus modulates the collective neural activity only weakly (in the order of $∼\alpha^{-3}$). In addition, neurons respond to the stimulus with some delay governed by the effective time constant $\tau_{n}$.

### 5.1. Mutual Information between Neural Activities and the Stimulus

The degree of correlations between the neural collective activity and the stimulus velocity is quantified by mutual information $I(\bar{r},v)$ as

(48) $$\begin{array}{c} I\left(\bar{r},v\right)={\langle \ln\rho \left(\bar{r}|v\right)\rangle }_{P(\bar{r},v)}-{\langle \ln\rho \left(\bar{r}\right)\rangle }_{\rho (\bar{r})}, \end{array}$$

where $\rho (\bar{r})$ is the distribution of neural activities, and averaging in the first term is performed over the joint probability density $P(\bar{r},v)$ of collective neural activity $\bar{r}$ and stimulus velocity *v*, with $P\left(\bar{r},v\right)=\rho \left(\bar{r}|v\right)P\left(v\right)$. The distribution $\rho (\bar{r})$ is found by marginalizing the joint distribution $P(\bar{r},v)$ over velocities *v*. The result of this procedure is (up to order $\alpha^{-3}$)

(49) $$\begin{array}{c} \rho \left(\bar{r},t\right)\approx \frac{\exp\left(-\frac{N}{2\sigma_{r}^{2}\left(t\right)}{\left[\bar{r}-r_{0}+\frac{r_{1}}{\alpha^{3}}\int_{0}^{t}dt^{\prime }e^{t^{\prime }/\tau_{n}}{\langle v^{2}\left(t^{\prime }\right)\rangle }_{P(v)}\right]}^{2}\right)}{\sqrt{2\pi \sigma_{r}^{2}\left(t\right)/N}}, \end{array}$$

where $r_{0}$ and $r_{1}$ are the stimulus-independent and the stimulus-dependent collective neural activities

(50) $$\begin{array}{c} r_{0}=\bar{r}\left(0\right)e^{-t/\tau_{n}}+\frac{\epsilon \kappa r_{m}}{\alpha }\left(1-e^{-t/\tau_{n}}\right),\\ r_{1}=\frac{\epsilon \kappa r_{m}}{2\tau_{n}}e^{-t/\tau_{n}}. \end{array}$$

Since both distributions $\rho (\bar{r})$ and $\rho (\bar{r}|v)$ are Gaussian, the mutual information $I(\bar{r},v)$ can be calculated easily as

(51) $$\begin{array}{c} I\left(\bar{r},v\right)\approx \frac{N{\left(\epsilon \kappa r_{m}\right)}^{2}}{8\alpha^{6}\sigma_{r}^{2}\left(t\right)}e^{-2t/\tau_{n}}\int_{0}^{t}dt_{1}\int_{0}^{t}dt_{2}e^{\left(t_{1}+t_{2}\right)/\tau_{n}}\\ \times \left[{\langle v^{2}\left(t_{1}\right)v^{2}\left(t_{2}\right)\rangle }_{P(v)}-{\langle v^{2}\left(t_{1}\right)\rangle }_{P(v)}{\langle v^{2}\left(t_{2}\right)\rangle }_{P(v)}\right]+O\left(\alpha^{-8}\right). \end{array}$$

This equation shows that the mutual information between neural collective activity and the stimulus velocity is proportional to the averaged temporal auto-correlations of the velocity square. Moreover, the larger the number of neurons *N* decoding the stimulus and the larger the network enhancement factor κ, the higher the mutual information. This clearly indicates that the effect of the network is a key ingredient for the accurate decoding of information from the outside world.

It is also interesting to see the effect of the time scale associated with variability in the stimulus velocity on the mutual information $I(\bar{r},v)$ in Equation (51). Assuming that the temporal auto-correlations of the stimulus velocity square are characterized by time constant $\tau_{c}$, i.e., that they decay exponentially as ${\langle v^{2}\left(t_{1}\right)v^{2}\left(t_{2}\right)\rangle }_{P(v)}-{\langle v^{2}\left(t_{1}\right)\rangle }_{P(v)}{\langle v^{2}\left(t_{2}\right)\rangle }_{P(v)}=C_{0}e^{-|t_{1}-t_{2}|/\tau_{c}}$, where $C_{0}$ is some constant, we find that (see Appendix B)

(52) $$\begin{array}{c} I{\left(\bar{r},v\right)}_{t\mapsto \infty }\approx \frac{N{\left(\epsilon \kappa r_{m}\right)}^{2}}{8\alpha^{6}\sigma_{r}^{2}}\frac{C_{0}\tau_{n}^{2}\tau_{c}}{\left(\tau_{n}+\tau_{c}\right)}+O\left(\alpha^{-8}\right). \end{array}$$

This implies that for very fast variability in the stimulus velocity ($\tau_{c}\mapsto 0$), the mutual information between the stimulus and the neural activity is close to 0. Consequently, neurons in this limit cannot track the particle velocity at all. However, as the stimulus variability slows down ($\tau_{c}$ grows), the mutual information increases and saturates at $\tau_{c}\gg \tau_{n}$. This means that in this limit, neurons can decode the stimulus optimally. In general, this result shows that time scale separation is important for the quality of neural inference, with a preference for slower stimuli, which agrees with the general results obtained in [88].

### 5.2. Energy Cost of Decoding the Stimulus

Guessing the actual value of the stimulus by neural network is not free of cost. In fact, it requires some amount of energy that neurons have to use to perform that function well. The energy used by neurons can be estimated by calculating the entropy production rate, with the help of Equation (21), with the probability density represented by a conditional distribution for collective neural activity given by Equation (46). We find the conditional entropy production rate ${\dot{S}}_{\rho (\bar{r}|v)}$ (conditioned on the stimulus velocity *v*) of neural activity as

(53) $$\begin{array}{c} {\dot{S}}_{\rho (\bar{r}|v)}=\frac{1}{\tau_{n}}\left(\frac{e^{-2t/\tau_{n}}}{\left(e^{2t/\tau_{n}}-1\right)}+\frac{N{\left[{\langle \bar{r}\left(t\right)\rangle }_{\rho (\bar{r}|v)}-\kappa \bar{c}\left(v\right)\right]}^{2}}{\sigma_{r}^{2}}\right). \end{array}$$

This formula indicates that the higher the discrepancy between the population-averaged tuning curve and the averaged neural activity, the larger the entropy production rate of neurons. In other words, neurons make an energetic effort to keep track of the actual particle velocity.

More explicit formula for the entropy production for longer times ($t\gg t_{n}$), after transients are gone, is

(54) $$\begin{array}{c} {\dot{S}}_{\rho (\bar{r}|v)}\approx \frac{N{\left(\epsilon \kappa r_{m}\right)}^{2}}{4\alpha^{6}\sigma_{r}^{2}\tau_{n}}{\left(v^{2}\left(t\right)-\frac{e^{-t/\tau_{n}}}{\tau_{n}}\int_{0}^{t}\;dt^{\prime }e^{t^{\prime }/\tau_{n}}{\langle v^{2}\left(t^{\prime }\right)\rangle }_{P(v)}\right)}^{2}, \end{array}$$

which implies that ${\dot{S}}_{\rho (\bar{r}|v)}$ is proportional to fluctuations of the square of velocity around its delayed average. Thus, for stationary stimulus velocity, its tracking by neurons is essentially energetically costless (neurons, however, use energy for other biophysical processes [5,6,7,13]). Note also that the prefactor in Equation (54) is the same as that in Equation (51) for the mutual information between neural activities and the stimulus velocity. This means that gaining information about the outside signals requires a proportionally large supply of energy; i.e., better prediction needs proportionally more energy.

## 6. Stochastic Dynamics of Synaptic Plasticity: Learning and Memory Storage

Synaptic weights are not fixed but change in the neural network although much slower than neural electric activities. Synaptic plasticity is the mechanism with which synaptic weights change, and it is responsible for learning and memory formation in neural systems [19,89,90,91,92]. The model analyzed in this section is a novel extension and modification of the model analyzed in [14].

### 6.1. Dynamics of Synaptic Weights

One of the most influential and important models of synaptic plasticity is the so-called BCM model [51], which was used for understanding the development of the mammalian visual cortex. It is an extension of the Hebb idea that connections between simultaneously activated presynaptic and postsynaptic neurons become stronger, but the model is constructed in such a way that the synaptic weights stabilize at some level without catastrophic run-away as it takes place for a classic Hebb’s rule [19]. The BCM plasticity rule, which was originally a deterministic rule, was extended to a stochastic rule by the author in [14], because synaptic plasticity is stochastic in nature [93,94]. In the case of a given postsynaptic neuron with activity *r*, which receives $N_{s}$ synaptic inputs from neurons with activities $f_{i}$ ($i=1,\ldots ,N_{s}$), the stochastic BCM rule takes the following form [14]

(55) $$\begin{array}{c} \frac{dw_{i}}{dt}=\lambda f_{i}r\left(r-\theta \right)-\frac{w_{i}}{\tau_{w}}+\frac{\sqrt{2}\sigma_{w}}{\sqrt{\tau_{w}}}\xi_{i} \end{array}$$

(56) $$\begin{array}{c} \tau_{\theta }\frac{d\theta }{dt}=-\theta +\beta r^{2}, \end{array}$$

where $w_{i}$ is the synaptic weight (proportional to the number of receptors on a synaptic membrane) related to the electric conductance of signals coming from presynaptic neuron *i*, λ is the amplitude of synaptic plasticity controlling the rate of change of synaptic weight, $\tau_{w}$ is the synaptic time constant controlling the weight decay duration, θ is the homeostatic variable, the so-called sliding threshold (adaptation for plasticity) related to an interplay of LTP and LTD (respectively, long-term potentiation and long-term depression [19]) with the time constant $\tau_{\theta }$, and β is the coupling intensity of θ to the postsynaptic firing rate *r*. The parameter $\sigma_{w}$ is the standard deviation of weights due to stochastic intrinsic fluctuations in synapses, which are represented as Gaussian white noise $\xi_{i}$ with zero mean and Delta function correlations, i.e., ${\langle \xi_{i}\left(t\right)\rangle }_{\eta }=0$ and ${\langle \xi_{i}\left(t\right)\xi_{j}\left(t^{\prime }\right)\rangle }_{\eta }=\delta_{ij}\delta \left(t-t^{\prime }\right)$ [53]. Equations (55) and (56) correspond to plastic synapses located on a single neuron. We consider this example, because it is easier to analyze than the whole network of neurons.

It is often assumed that $\tau_{\theta }/\tau_{w}\ll 1$, and then the homeostatic variable achieves a steady state on the time scale for changes in synaptic weights, i.e., dθ/dt≈0. This means that for long times, we have approximately $\theta \approx \beta r^{2}$, and consequently, the BCM rule takes a simple (one equation) form

(57) $$\begin{array}{c} \frac{dw_{i}}{dt}=\lambda f_{i}r^{2}\left(1-\beta r\right)-\frac{w_{i}}{\tau_{w}}+\frac{\sqrt{2}\sigma_{w}}{\sqrt{\tau_{w}}}\xi_{i}. \end{array}$$

As we saw in the previous section, the neural network function is determined primarily by the collective dynamics of neurons and synapses. For that reason, it makes sense to consider also the dynamics of the population-averaged synaptic weight. In this case, it is not the population average of all synapses in the network but rather the population average of synapses on a single neuron, i.e., $\bar{w}=\left(1/N_{s}\right)\sum_{i}w_{i}$. Summing both sides of Equation (57) with the rescaling factor $N_{s}$, we obtain the population averaged dynamics of $\bar{w}$

(58) $$\begin{array}{c} \frac{d\bar{w}}{dt}=\lambda \bar{f}r^{2}\left(1-\beta r\right)-\frac{\bar{w}}{\tau_{w}}+\frac{\sqrt{2}\sigma_{w}}{\sqrt{N_{s}\tau_{w}}}\bar{\xi }, \end{array}$$

where $\bar{f}=\left(1/N_{s}\right)\sum_{i}f_{i}$, and $\bar{\xi }=\left(1/\sqrt{N_{s}}\right)\sum_{i}\xi_{i}$. Moreover, the neural activity is much faster than the synaptic dynamics (seconds vs. minutes), i.e., $\tau_{n0}/\tau_{w}\ll 1$. Hence, the neural dynamics also reach a quasi-stationary state on the time scales $∼\tau_{w}$, and it can be approximated by (from Equation (41))

(59) $$\begin{array}{c} r\approx c\left(v\right)+\tau_{n0}\bar{f}\bar{w}, \end{array}$$

where we used a mean-field expression $\left(1/N_{s}\right)\sum_{i}w_{i}f_{i}\approx \bar{w}\bar{f}$, and c(v) is given by Equation (42). In the following, we treat $\bar{f}$ as the time-independent fixed parameter characterizing the level of activity in the local network.

Inserting Equation (59) into Equation (58), we obtain an effective equation for the dynamics of population mean synaptic weight $\bar{w}$

(60) $$\begin{array}{c} \frac{d\bar{w}}{dt}=\lambda \bar{f}{\left[c\left(v\right)+\tau_{n0}\bar{f}\bar{w}\right]}^{2}\left(1-\beta [c\left(v\right)+\tau_{n0}\bar{f}\bar{w}]\right)-\frac{\bar{w}}{\tau_{w}}+\frac{\sqrt{2}\sigma_{w}}{\sqrt{N_{s}\tau_{w}}}\bar{\xi }, \end{array}$$

which has a general form of the Langevin equation as in Equation (2) with the generalized force acting on synapses

(61) $$\begin{array}{c} F_{w}\left(\bar{w}\right)=\lambda \bar{f}{\left[c\left(v\right)+\tau_{n0}\bar{f}\bar{w}\right]}^{2}\left(1-\beta [c\left(v\right)+\tau_{n0}\bar{f}\bar{w}]\right)-\frac{\bar{w}}{\tau_{w}}. \end{array}$$

That force depends nonlinearly on $\bar{w}$, which is one of the reasons for complex dynamics of synaptic plasticity, which are additionally influenced by synaptic noise (∼$\sigma_{w}$). Note, however, that the noise for the mean synaptic weight is much weaker than the noise in individual synapses due to the rescaling factor $1/\sqrt{N_{s}}$.

How do synaptic weights react to the sensory input represented by the tuning curve c(v) (see Equation (42))? Since we consider here a single postsynaptic neuron, and it has a preferred velocity of the stimulus that is mostly different than the actual velocity of the stimulus, the value of c(v) is most of the time close to zero. The stimulus c(v) jumps between 0 and its maximal value $r_{m}$ only transiently at precisely those times when the velocity of the outside particle matches the preferred velocity of the neuron. This is the basic setup we consider here: input coming to synapses is transient, which however can be enough to increase significantly their mean population weight $\bar{w}$ in some circumstances. This process of changing $\bar{w}$ is essentially the “learning” information about the particle velocity, which can be stored in the mean weight $\bar{w}$ for some time (“memory”). Below, we describe in more detail how these two processes, learning and memory, take place within this model.

Given the transient nature of c(v), we consider it as a perturbation to the collective synaptic dynamics in Equation (60). The deterministic version of Equation (60), i.e., with $\sigma_{w}=0$, for c(v)=0, can have either one fixed point at $\bar{w}=0$, or three fixed points, of which two are stable, corresponding to bistability (the fixed points are the solutions of the equation $F_{w}=0$). The change from monostability to bistability in the dynamics takes place if the following condition is satisfied:

(62) $$\begin{array}{c} \lambda \tau_{w}\tau_{no}{\bar{f}}^{2}>4\beta , \end{array}$$

which happens for sufficiently large plasticity amplitude λ and/or presynaptic firing rate $\bar{f}$. In the bistable regime, the two stable fixed points are denoted as ${\bar{w}}_{d}$ (“down” state) and ${\bar{w}}_{u}$ (“up” state), and they have the following values:

(63) $$\begin{array}{c} {\bar{w}}_{d}=0,\\ {\bar{w}}_{u}=\frac{1+\sqrt{1-\beta_{f}}}{2\beta \tau_{n0}\bar{f}}, \end{array}$$

where $\beta_{f}=4\beta /\left(\lambda \tau_{w}\tau_{n0}{\bar{f}}^{2}\right)$. The unstable fixed point denoted as ${\bar{w}}_{m}$ (middle state) is

(64) $$\begin{array}{c} {\bar{w}}_{m}=\frac{1-\sqrt{1-\beta_{f}}}{2\beta \tau_{n0}\bar{f}}. \end{array}$$

Note that ${\bar{w}}_{u}$ and ${\bar{w}}_{m}$ are pushed toward zero for very large presynaptic firing rates $\bar{f}$, which suggests that bistability is lost for very large presynaptic firing $\bar{f}$.

**Figure 1:**
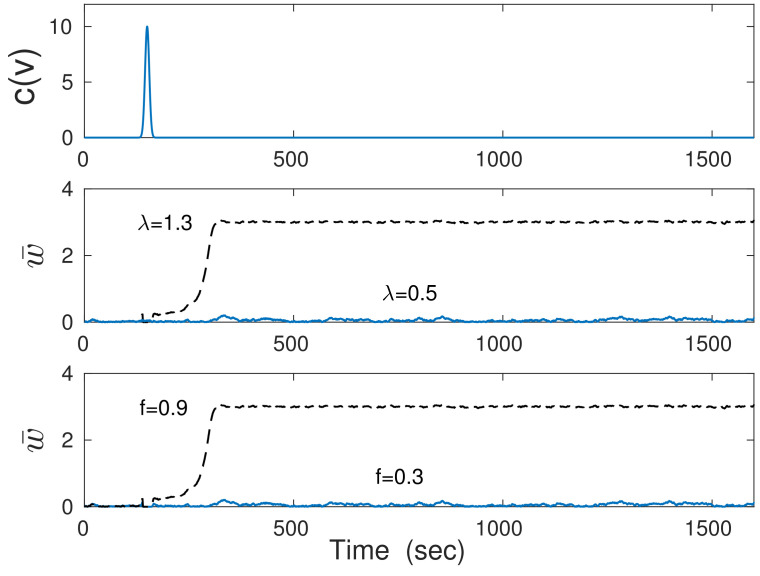
**Stimulus-induced transition from weak to strong synapses.** Transient input c(v) to the neuron can induce a transition in the collective weight of synapses $\bar{w}$ (upper panel). Transitions from weak ($\bar{w}\approx {\bar{w}}_{d}$) to strong ($\bar{w}\approx {\bar{w}}_{u}$) synapses take place only when the amplitude of synaptic plasticity λ or firing rate of presynaptic neurons $\bar{f}$ are sufficiently large (middle and lower panels). Note that $\bar{w}$ can maintain the value ${\bar{w}}_{u}$ for a very long time, much larger than the synaptic time constant $\tau_{w}=200$ s (synaptic memory trace about *c*), because collective stochastic fluctuations are rescaled by the number of synapses $1/\sqrt{N_{s}}$. The middle and lower panels look almost identical despite different parameters, because the noise term in Equation (60) dominates for most of the time in this regime. The nominal parameters used are λ=1.3, β=1.2, $\bar{f}=0.9$ Hz, $\tau_{n}=0.3$ s, $\tau_{w}=200$ s, $\sigma_{w}=5.0$, $N_{s}=1000$, $r_{m}=10$ Hz, u=10 mm/s, ϵ=0.1 mm/s. In this example, the stimulus moves with the linearly increasing velocity v=0.02t+7 (mm/s) with a small accelaration of 0.02 mm/s^2^. Too large accelaration prohibits the synaptic transition to the state with ${\bar{w}}_{u}$.

Now, consider the stochastic version of Equation (60), i.e., with inclusion of the noise ($\sigma_{w}\neq 0$). In this case, the brief input c(v) can cause a dynamic transition from the down state (${\bar{w}}_{d}$) to the up state (${\bar{w}}_{u}$) in the collective behavior of synapses but only if two conditions are met (Figure 1). The first is the bistability condition represented by Equation (62). The second condition is such that the input c(v) cannot be too brief, which translates to the requirement that the stimulus velocity cannot change too quickly. The latter simply means that slow synapses are unable to react to too-fast inputs (Figure 1), which is a similar situation to the case of poor neural inference of too-fast stimuli (see, the previous Section). The successful transition to the up state ${\bar{w}}_{u}$ is a form of brief learning, and maintaining the acquired information about the stimulus *c* for a long time represents the memory trace. Keeping the information in the synaptic weights for a prolonged time is possible even for very strong intrinsic noise ($\sigma_{w}∼{\bar{w}}_{u}$), because collective synaptic noise is suppressed by the number of synapses $N_{s}$ (compare Equations (57) and (60)). Ultimately, the memory will be lost, i.e., $\bar{w}$ will decay from ${\bar{w}}_{u}$ to ${\bar{w}}_{d}$, and this can happen in several ways. The most likely are a very strong downward noise fluctuation or a significant drop in the presynaptic activity $\bar{f}$ below some level.

Instead of speaking about forces acting on synapses, we can alternatively say that the population mean of synaptic weight moves in an effective potential $V(\bar{w},c)$, given by $V\left(\bar{w},c\right)=-\int_{0}^{\bar{w}}\;dxF_{w}\left(x,c\right)$. It can be determined explicitly, and it is composed of two contributions

(65) $$\begin{array}{c} V\left(\bar{w},c\right)=V_{0}\left(\bar{w}\right)+\Delta V\left(\bar{w},c\right), \end{array}$$

where $V_{0}\left(\bar{w}\right)$ is the “core” potential

(66) $$\begin{array}{c} V_{0}\left(\bar{w}\right)=\frac{{\bar{w}}^{2}}{2\tau_{w}}-\frac{1}{3}\lambda {\bar{f}}^{3}\tau_{n0}^{2}{\bar{w}}^{3}+\frac{1}{4}\lambda {\bar{f}}^{4}\tau_{n0}^{3}\beta {\bar{w}}^{4}, \end{array}$$

and $\Delta V(\bar{w},c)$ is the perturbation to the core potential due to the transient stimulus

(67) $$\begin{array}{c} \Delta V\left(\bar{w},c\right)=-\lambda \bar{f}c^{2}\left(1-\beta c\right)\bar{w}-\frac{1}{2}\lambda {\bar{f}}^{2}\tau_{n0}c\left(2-3\beta c\right){\bar{w}}^{2}+\lambda {\bar{f}}^{3}\tau_{n0}^{2}\beta c{\bar{w}}^{3}. \end{array}$$

**Figure 2:**
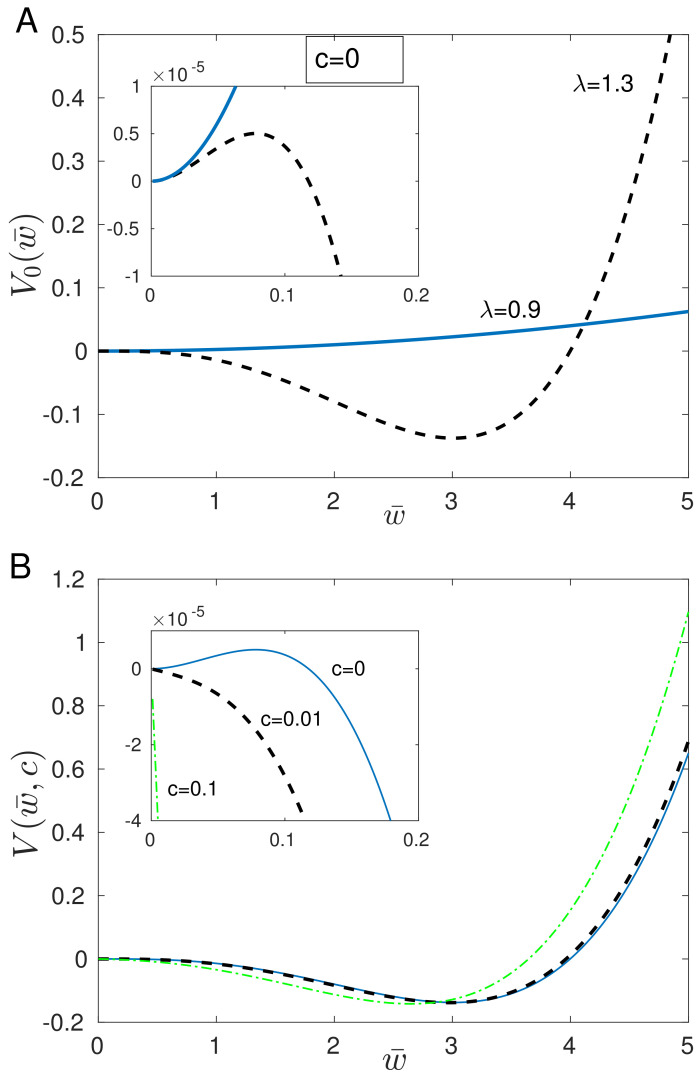
**Effective potential $V(\bar{w},c)$ for the collective synaptic weights and bistability.** (**A**) The core potential $V_{0}\left(\bar{w}\right)$ has either one minimum, for sufficiently weak plasticity amplitude λ, or two minima for stronger λ. The latter corresponds to bistability in the collective behavior of synapses. Note that the miniumum at $\bar{w}=0$ is very shallow (inset). (**B**) The bistability regime. The presence of even a weak stimulus c(v) lowers the potential barrier in $V(\bar{w},c)$ between the shallow and the deep minima, which can facilitate a transition from weak to strong synapses ($\bar{w}$ can change from ${\bar{w}}_{d}$ to ${\bar{w}}_{u}$). The parameters used are the same as in Figure 1.

The core potential $V_{0}\left(\bar{w}\right)$ can have either one minimum (monostability) or two minima (bistability) depending on the strength of synaptic plasticity λ and/or the level of presynaptic neural activity $\bar{f}$ (Figure 2A). The “phase transition” from monostability to bistability occurs if the condition in Equation (62) is satisfied, which is the same as the condition for the appearance of the three fixed points. Thus, one can think about the plasticity amplitude λ or the presynaptic firing rate $\bar{f}$ as tuning parameters for the phase transition in this model. More interesting for information storing is the bistable regime with two minima, as is the case with storing information in electronic hardware [26,33], and we focus on this case below. The minima of $V_{0}$ are situated exactly at the two stable fixed points ${\bar{w}}_{d}$ and ${\bar{w}}_{u}$ determined before (Equation (63)). The maximum of $V_{0}$ appears at the middle (unstable) fixed point ${\bar{w}}_{m}$. However, note that for the realistic synaptic and neural parameters, the minimum at ${\bar{w}}_{d}$ is very shallow (Figure 2A), and this is due to the large synaptic time constant $\tau_{w}$.

In the potential-like picture, the effective mean synaptic weight wanders around the two minima of the potential $V_{0}\left(\bar{w}\right)$ with occasional large jumps over the potential barrier (i.e., the maximum) triggered either by turning on the input c(v), or by noise, or both (Figure 2B). However, the transitions from ${\bar{w}}_{d}$ to ${\bar{w}}_{u}$ are more easier and frequent than the reverse transitions due to the shallowness of the potential $V_{0}$ at ${\bar{w}}_{d}$. This means that not only sensory input can trigger the learning and subsequent “memory” of that input but also the noise can induce sporadically “learning and memory”. The latter can be thought as false memories, which are also present in real brains.

The dwelling times of the collective weight $\bar{w}$ close to the minima at ${\bar{w}}_{d}$ and ${\bar{w}}_{u}$ can be found from the well-known Kramers’ formula [53]. In our case, they are given by

(68) $$\begin{array}{c} T_{d}=\frac{2\pi }{\sqrt{V_{d}^{\left(2\right)}\left|V_{m}^{\left(2\right)}\right|}}\exp\left(\frac{N_{s}\tau_{w}}{\sigma_{w}^{2}}\left[\left(V_{0,m}-V_{0,d}\right)+\left(\Delta V_{m}-\Delta V_{d}\right)\right]\right),\\ T_{u}=\frac{2\pi }{\sqrt{V_{u}^{\left(2\right)}\left|V_{m}^{\left(2\right)}\right|}}\exp\left(\frac{N_{s}\tau_{w}}{\sigma_{w}^{2}}\left[\left(V_{0,m}-V_{0,u}\right)+\left(\Delta V_{m}-\Delta V_{u}\right)\right]\right), \end{array}$$

where $V_{0,m}=V_{0}\left({\bar{w}}_{m}\right)$, $V_{0,d}=V_{0}\left({\bar{w}}_{d}\right)$, and $V_{0,u}=V_{0}\left({\bar{w}}_{u}\right)$, and analogically for ΔV. (Note that $V_{0,d}=\Delta V_{d}=0$.) The quantity in the exponent of $T_{d}$ ($T_{u}$) is proportional to the potential barrier between the minimum at ${\bar{w}}_{d}$ (${\bar{w}}_{u}$) and the maximum at ${\bar{w}}_{m}$. The symbols $V_{d}^{\left(2\right)},V_{u}^{\left(2\right)},V_{m}^{\left(2\right)}$ denote the second derivatives of $V(\bar{w},c)$ with respect to $\bar{w}$ at points ${\bar{w}}_{d}$, ${\bar{w}}_{u}$, and ${\bar{w}}_{m}$, respectively. The formulas in Equation (68) indicate that switching on the input *c* causes the deformation of the potential barrier (Figure 2B). In particular, in our case $\Delta V_{m}-\Delta V_{d}<0$ for c>0, meaning that the barrier from the down to up state decreases, which can facilitate the transition to the up state if synapses were initially in the lower state. Moreover, while the fluctuations around the minima are much slower than neural activity ($\tau_{n0}$), they are more frequent ($∼\tau_{w}$) than the jumps over the potential barrier, which happen rarely ($∼T_{d},T_{u}\gg \tau_{w}$).

The existence of bistability in the collective behavior of synapses implies that we can effectively represent the continuous stochastic dynamics of synaptic weights as the jumping dynamics of a two-state system. In this discrete effective system, we can define probability $p_{d}$ that the collective state of all $N_{s}$ synapses has the weight ${\bar{w}}_{d}$ and another probability $p_{u}$ corresponding to the higher population weight ${\bar{w}}_{u}$. The transition rates between the down and up states can be determined from the dwelling times as their inverses. In particular, the transition rate $\omega_{ud}$ from the down to up state is $\omega_{ud}=1/T_{d}$, and the opposite transition the from up to down state is $\omega_{du}=1/T_{u}$. In our case, because of the asymmetric potential, we have that $\omega_{du}\ll \omega_{ud}$, i.e., the transitions to the up state are more frequent than in the opposite direction. The master equation associated with this dynamic is

(69) $$\begin{array}{c} {\dot{p}}_{u}=\omega_{ud}\left(1-p_{u}\right)-\omega_{du}p_{u}, \end{array}$$

and $p_{d}=1-p_{u}$. From the above, it is clear that the transition rates $\omega_{ud},\omega_{du}$ are approximately the products of two terms: $\omega_{ud}=\omega_{ud,0}\Gamma_{ud}\left(c\right)$ and $\omega_{du}=\omega_{du,0}\Gamma_{du}\left(c\right)$, one of which is independent of the input *c* ($\omega_{ud,0}$ and $\omega_{du,0}$) and the second is dependent on it via ΔV (the terms $\Gamma_{ud}\left(c\right)$ and $\Gamma_{du}\left(c\right)$). Thus, turning on the input can modify the distribution of the probabilities $p_{d},p_{u}$, and it can also induce transitions. The existence of bistability for the population of synapses can be also useful in terms of information storing, which we address next.

### 6.2. Information Gain and Maintenance, and Associated Energy Cost

Learning in our synaptic system can be thought as gaining information about the stimulus *c* due to its brief switching on and off. Such a transient change causes changes in $\omega_{du},\omega_{ud}$, which modifies the probabilities $p_{d},p_{u}$. The information gain can be quantified by calculating the KL divergence between an initial distribution of probabilities after the brief learning and the final steady-state distribution. Memory in this system can be thought as maintaining that information for a prolonged time after the stimulus *c* was brought to 0.

Below, we consider in detail the maintenance of the information, and its associated energy cost, and this is a novel analysis. Let us assume that at time t=0, the collective synaptic system has a probability $p_{u}\left(0\right)$ larger than its steady-state value (before learning) $p_{u,\infty }=\omega_{ud,0}/\omega_{0}$, where $\omega_{0}=\omega_{du,0}+\omega_{ud,0}$. At t=0 the stimulus is switched off and the transition rates suddenly jump to their steady state values ($\omega_{ud}\mapsto \omega_{ud,0}$, and $\omega_{du}\mapsto \omega_{du,0}$). Consequently, the probability $p_{u}\left(t\right)$ relaxes to its steady-state value $p_{u,\infty }$ according to $p_{u}\left(t\right)=\left[p_{u}\left(0\right)-p_{u,\infty }\right]e^{-\omega_{0}t}+p_{u,\infty }$. This relaxation is related to losing the acquired information during the learning phase and has a characteristic time scale, which in this case can be called the memory lifetime $T_{m}=1/\omega_{0}$. Thus, the memory lifetime is equivalent to a temporal retaining of information about the stimulus in the population of synaptic weights.

The loss of information about the stimulus can be also quantified by the KL divergence $D_{KL}(\vec{p}\left(t\right)||{\vec{p}}_{\infty })$ between the actual probability distribution $\vec{p}\left(t\right)=\left(p_{d}\left(t\right),p_{u}\left(t\right)\right)$ and the steady-state distribution ${\vec{p}}_{\infty }=\left(p_{d,\infty },p_{u,\infty }\right)$. We find

(70) $$\begin{array}{c} D_{KL}(\vec{p}\left(t\right)||{\vec{p}}_{\infty })=\left[p_{d,\infty }-\Delta e^{-\omega_{0}t}\right]\ln\left(1-\left(\Delta /p_{d,\infty }\right)e^{-\omega_{0}t}\right)\\ +\left[p_{u,\infty }+\Delta e^{-\omega_{0}t}\right]\ln\left(1+\left(\Delta /p_{u,\infty }\right)e^{-\omega_{0}t}\right), \end{array}$$

where Δ characterizes the magnitude of an initial perturbation from the steady state caused by the transient stimulus, i.e., $\Delta =p_{u}\left(0\right)-p_{u,\infty }$, and Δ>0.

The rate of Kullback–Leibler divergence, denoted as ${\dot{D}}_{KL}$, takes the form

(71) $$\begin{array}{c} {\dot{D}}_{KL}(\vec{p}\left(t\right)||{\vec{p}}_{\infty })=-\omega_{0}\Delta e^{-\omega_{0}t}\ln\left(\frac{1+\left(\Delta /p_{u,\infty }\right)e^{-\omega_{0}t}}{1-\left(\Delta /p_{d,\infty }\right)e^{-\omega_{0}t}}\right), \end{array}$$

from which it is clear that information is lost exponentially with the rate proportional to the inverse of memory lifetime $\omega_{0}$.

The energy loss during the relaxation to the steady state is proportional to the entropy production rate ${\dot{S}}_{w}$ in the synaptic weights. The latter is found from Equation (18) and yields

(72) $$\begin{array}{c} {\dot{S}}_{w}=-{\dot{D}}_{KL}(\vec{p}\left(t\right)||{\vec{p}}_{\infty }), \end{array}$$

which means that the entropy production rate increases precisely in such a way as to balance the decreasing rate of acquired information, i.e., ${\dot{D}}_{KL}$. The inverse relationship between ${\dot{S}}_{w}$ and memory lifetime ($\dot{S}∼\omega_{0}$) implies that the longer the information is retained, the smaller the rate of dissipated energy. This, in turn, suggests that the total entropy produced during the weights relaxation process, i.e., $S_{w,tot}=\int_{0}^{\infty }\;dt{\dot{S}}_{w}$, should be independent of memory lifetime. Indeed, we find

(73) $$\begin{array}{c} S_{w,tot}=p_{d}\left(0\right)\ln\left(\frac{p_{d}\left(0\right)}{p_{d,\infty }}\right)+p_{u}\left(0\right)\ln\left(\frac{p_{u}\left(0\right)}{p_{u,\infty }}\right), \end{array}$$

which means the total entropy produced is related in a simple way to the KL divergence between $\vec{p}\left(0\right)$ and ${\vec{p}}_{\infty }$, namely

(74) $$\begin{array}{c} S_{w,tot}=D_{KL}(\vec{p}\left(0\right)||{\vec{p}}_{\infty }). \end{array}$$

This equation can be interpreted in the following way: the energy cost associated with storing information in synapses is proportional to the discrepancy between the distribution of initially perturbed synaptic weights and their steady-state distribution. In general, Equations (72) and (74) indicate that the information-like quantity, which is $D_{KL}$, is closely related to the energy-like quantity ${\dot{S}}_{w}$. This is in line with the considerations in the previous sections about stochastic thermodynamics.

## 7. More General Framework for Synaptic Learning and Memory

The above approach for synaptic plasticity and learning may seem too simplistic. After all, representing different patterns of synaptic weights by a single collective variable $\bar{w}$ is probably too drastic, since by doing that, we throw out a lot of information about different synaptic states. An alternative approach is possible, and it is briefly described below. The details can be found in [18].

Here, we consider $N_{s}$ mutually coupled excitatory synapses on a single neuron (we assume that the neuron has a single dendrite along which synapses are linearly located). Each synapse can be in *K* discrete states $s_{i}=1,\ldots ,K$, where *i* denotes the synapse number. These states correspond to the different shapes and sizes of the postsynaptic part of a synapse called the dendritic spine, which can be regarded as mesoscopic well-defined morphological synaptic states, where microscopic (molecular) details are neglected [95,96]. It is hypothesized in the neuroscience community that these morphological states have functional roles, e.g., large synapses (spines) are slow and involved in storing long-term information (memory), while smaller synapses are fast and take part in acquiring information (learning) [90,97]. Moreover, the states with small values of $s_{i}$ correspond to weaker synaptic weights (a smaller number of molecular receptors on the synapse membrane), and larger values of $s_{i}$ correspond to stronger synaptic weights.

Let $P(\vec{s})$ be the probability that these synapses are in the global state described by the vector $\vec{s}=\left(s_{1},s_{2},\ldots ,s_{N}\right)$. The most general form of the master equation for the stochastic dynamics of $P(\vec{s})$ is

(75) $$\begin{array}{c} \frac{dP(\vec{s})}{dt}=\sum_{i=1}^{N_{s}}\sum_{s_{i}^{\prime }}\left[w_{s_{i},s_{i}^{\prime }}\left(s_{i-1},s_{i+1}\right)P\left({\vec{s}}_{i}^{\prime }\right)-w_{s_{i}^{\prime },s_{i}}\left(s_{i-1},s_{i+1}\right)P\left(\vec{s}\right)\right], \end{array}$$

where ${\vec{s}}_{i}^{\prime }=\left(s_{1},\ldots ,s_{i-1},s_{i}^{\prime },s_{i+1},\ldots ,s_{N}\right)$, and $w_{s_{i},s_{i}^{\prime }}\left(s_{i-1},s_{i+1}\right)$ is the transition rate for the jumps inside synapse *i* from state $s_{i}^{\prime }$ to state $s_{i}$. In agreement with the experimental data, these jumps also depend on the states of neighboring synapses $s_{i-1}$ and $s_{i+1}$ [97], and such synaptic cooperativity can be also useful for long-term memory stability [98,99,100]. The transition rates $w_{s_{i},s_{i}^{\prime }}\left(s_{i-1},s_{i+1}\right)$ can be composed of several different terms, each representing a different type of synaptic plasticity (e.g., hebbian, homeostatic) [101]. Additionally, each term can depend in a complicated manner on presynpatic and postsynaptic neural activities. It is also useful to note that Equation (75) is structurally similar to the Glauber dynamics for a time-dependent Ising model, which is known from statistical physics [52].

Unfortunately, Equation (75) is practically unsolvable for a large number of synapses $N_{s}$, because we have $K^{N_{s}}$ coupled differential equations to solve. For example, for K=2 and $N_{s}=1000$, we have $10^{100}$ equations, which is impossible to handle on any existing computer (more equations than the number of protons in the visible universe!). An useful approximation to these types of problems is provided by the so-called “pair approximation” [18]. The essence of this method lies in reducing the effective dimensionality of the synaptic system by considering only dynamics of single-synapse probabilities $P(s_{i})$ and double-synapse probabilities $P(s_{i},s_{i+1})$. This means that three-synapse correlations as well as higher-order correlations are neglected, which is in agreement with an intuition, since the coupling between synapses takes place between the nearest neighbors. In the pair approximation, the joint probability $P(\vec{s})$ is approximated as [18]

(76) $$\begin{array}{c} P\left(\vec{s}\right)\approx \frac{P\left(s_{1},s_{2}\right)\ldots P\left(s_{i-1},s_{i}\right)\ldots P\left(s_{N-1},s_{N}\right)}{P\left(s_{2}\right)\ldots P\left(s_{i}\right)\ldots P\left(s_{N-1}\right)}. \end{array}$$

This allows us to write the dynamics of probabilities $P(s_{i})$ and $P(s_{i},s_{i+1})$, which we obtain by marginalization of the joint probability $P(\vec{s})$, in the form

(77) $$\begin{array}{c} \frac{dP(s_{i})}{dt}\approx \sum_{s_{i-1}}\sum_{s_{i+1}}\sum_{s_{i}^{\prime }}[w_{s_{i},s_{i}^{\prime }}\left(s_{i-1},s_{i+1}\right)\frac{P\left(s_{i-1},s_{i}^{\prime }\right)P\left(s_{i}^{\prime },s_{i+1}\right)}{P(s_{i}^{\prime })}\\ -w_{s_{i}^{\prime },s_{i}}\left(s_{i-1},s_{i+1}\right)\frac{P\left(s_{i-1},s_{i}\right)P\left(s_{i},s_{i+1}\right)}{P(s_{i})}] \end{array}$$

for $i=2,\ldots ,N_{s}-1$, and

(78) $$\begin{array}{c} \frac{dP(s_{i},s_{i+1})}{dt}\approx \sum_{s_{i-1}}\sum_{s_{i}^{\prime }}[w_{s_{i},s_{i}^{\prime }}\left(s_{i-1},s_{i+1}\right)\frac{P\left(s_{i-1},s_{i}^{\prime }\right)P\left(s_{i}^{\prime },s_{i+1}\right)}{P(s_{i}^{\prime })}\\ -w_{s_{i}^{\prime },s_{i}}\left(s_{i-1},s_{i+1}\right)\frac{P\left(s_{i-1},s_{i}\right)P\left(s_{i},s_{i+1}\right)}{P(s_{i})}]\\ +\sum_{s_{i+2}}\sum_{s_{i+1}^{\prime }}[w_{s_{i+1},s_{i+1}^{\prime }}\left(s_{i},s_{i+2}\right)\frac{P\left(s_{i},s_{i+1}^{\prime }\right)P\left(s_{i+1}^{\prime },s_{i+2}\right)}{P(s_{i+1}^{\prime })}\\ -w_{s_{i+1}^{\prime },s_{i+1}}\left(s_{i},s_{i+2}\right)\frac{P\left(s_{i},s_{i+1}\right)P\left(s_{i+1},s_{i+2}\right)}{P(s_{i+1})}], \end{array}$$

for $i=2,\ldots ,N_{s}-2$. Similar expressions can be written for the boundary probabilities with i=1 and $i=N_{s}$.

Equations (77) and (78) form a closed system of differential equations. Most importantly, we have now only a $K\left(K+1\right)N_{s}/2$ equation to solve instead of $K^{N_{s}}$. This means that after applying the pair approximation, the computational complexity of the problem grows only linearly with the number of synapses $N_{s}$, not exponentially. The solution of the system given by Equations (77) and (78) allows us to determine information gain and its energy cost during synaptic learning (during the LTP phase).

### Information Gain and Loss, and Associated Energy Cost

Let us assume that before learning, synapses have a steady-state distribution $P_{ss}\left(\vec{s}\right)$. Learning causes modifications in synaptic structures, which are associated with modified transition rates and non-equilibrium jumps between different states. As before, KL divergence can be used to quantify information gain during the learning phase (Equation (14)), which in our case takes the form

(79) $$\begin{array}{c} D_{KL}(P\left(\vec{s}\right)\left|\right|P_{ss}\left(\vec{s}\right))=\sum_{\vec{s}}P\left(\vec{s}\right)\ln\frac{P(\vec{s})}{P_{ss}\left(\vec{s}\right)}. \end{array}$$

The temporal rate of gaining information during LTP can be found with the help of the above pair approximation as

(80) $$\begin{array}{c} {\dot{D}}_{KL}(P\left(\vec{s}\right)\left|\right|P_{ss}\left(\vec{s}\right))\approx \sum_{s_{1},s_{1}^{\prime }}\sum_{s_{2}}\left[w_{s_{1},s_{1}^{\prime }}\left(s_{2}\right)P\left(s_{1}^{\prime },s_{2}\right)-w_{s_{1}^{\prime },s_{1}}\left(s_{2}\right)P\left(s_{1},s_{2}\right)\right]\ln\frac{P(s_{1},s_{2})}{P_{ss}\left(s_{1},s_{2}\right)}\\ +\sum_{s_{N_{s}},s_{N_{s}}^{\prime }}\sum_{s_{N_{s}-1}}\left[w_{s_{N_{s}},s_{N_{s}}^{\prime }}\left(s_{N_{s}-1}\right)P\left(s_{N_{s}-1},s_{N_{s}}^{\prime }\right)-w_{s_{N_{s}}^{\prime },s_{N_{s}}}\left(s_{N_{s}-1}\right)P\left(s_{N_{s}-1},s_{N_{s}}\right)\right]\ln\frac{P(s_{N_{s}-1},s_{N_{s}})}{P_{ss}\left(s_{N_{s}-1},s_{N_{s}}\right)}\\ +\sum_{i=2}^{N_{s}-1}\sum_{s_{i},s_{i}^{\prime }}\sum_{s_{i-1},s_{i+1}}[w_{s_{i},s_{i}^{\prime }}\left(s_{i-1},s_{i+1}\right)\frac{P\left(s_{i-1},s_{i}^{\prime }\right)P\left(s_{i}^{\prime },s_{i+1}\right)}{P(s_{i}^{\prime })}\\ -w_{s_{i}^{\prime },s_{i}}\left(s_{i-1},s_{i+1}\right)\frac{P\left(s_{i-1},s_{i}\right)P\left(s_{i},s_{i+1}\right)}{P(s_{i})}]\ln\frac{P\left(s_{i-1},s_{i}\right)P\left(s_{i},s_{i+1}\right)P_{ss}\left(s_{i}\right)}{P_{ss}\left(s_{i-1},s_{i}\right)P_{ss}\left(s_{i},s_{i+1}\right)P\left(s_{i}\right)} \end{array}$$

Thus, ${\dot{D}}_{KL}$ depends on the transition rates between synaptic states, which is similar to the entropy production rate related to the energy cost of synaptic plasticity.

The entropy production rate of synaptic transitions in this approximation is

(81) $$\begin{array}{c} {\dot{S}}_{pr}\left(\vec{s}\right)=\sum_{i=1}^{N_{s}}{\dot{S}}_{pr,i} \end{array}$$

where ${\dot{S}}_{pr,i}$ is the individual entropy production in synapse *i*, which is

(82) $$\begin{array}{c} {\dot{S}}_{pr,i}\approx \frac{1}{2}\sum_{s_{i-1},s_{i+1}}\sum_{s_{i},s_{i}^{\prime }}[w_{s_{i},s_{i}^{\prime }}\left(s_{i-1},s_{i+1}\right)\frac{P\left(s_{i-1},s_{i}^{\prime }\right)P\left(s_{i}^{\prime },s_{i+1}\right)}{P(s_{i}^{\prime })}\\ -w_{s_{i}^{\prime },s_{i}}\left(s_{i-1},s_{i+1}\right)\frac{P\left(s_{i-1},s_{i}\right)P\left(s_{i},s_{i+1}\right)}{P(s_{i})}]\\ \times \ln\frac{w_{s_{i},s_{i}^{\prime }}\left(s_{i-1},s_{i+1}\right)P\left(s_{i-1},s_{i}^{\prime }\right)P\left(s_{i}^{\prime },s_{i+1}\right)P\left(s_{i}\right)}{w_{s_{i}^{\prime },s_{i}}\left(s_{i-1},s_{i+1}\right)P\left(s_{i-1},s_{i}\right)P\left(s_{i},s_{i+1}\right)P\left(s_{i}^{\prime }\right)}. \end{array}$$

The physical energy cost of synaptic plasticity is $∼E_{0}{\dot{S}}_{pr}\left(\vec{s}\right)$, where $E_{0}$ is the energy scale associated with plasticity processes in a single synapse (for details, see [14,18]). In general, $E_{0}∼10^{5}k_{B}T$, since a synapse, although small, is a composite object consisting of many different molecular degrees of freedom [14].

As can be seen, both Equations (80) and (82) have a similar structure, suggesting that the information gain rate and its energy requirement depend similarly on time, and they are generally proportional to one another. This means acquiring larger information during learning incurs higher energy costs, which is mainly because of the prefactor $E_{0}$. Again, information is physical and costly. Moreover, the cooperativity between neighboring synapses (reflected in the transition rates $w_{s_{i},s_{i}^{\prime }}\left(s_{i-1},s_{i+1}\right)$) can have a positive effect on energy efficiency of information gain if synapses are positively correlated [18].

## 8. Concluding Remarks

Basic components of the brain, i.e., neurons and synapses, exhibit probabilistic behavior because they are affected by noisy internal and external signals [87]. In this paper, the goal was to show that the concepts of information thermodynamics can be useful in neuroscience problems, in which there is inherent stochasticity. Such problems involve neural inference as well as synaptic learning and memory. In all these neurobiological examples, neurons and synapses handle information, and since information is physical, the brain has to use some amount of energy while executing its computations [4,5,13,14,18]. If we assume that the brain uses information economically (e.g., [29]), then not all of these computations are equally likely. Consequently, knowing the probability of a given neural or synaptic activity (for a given task) should be a crucial element in deciphering the rules governing brain computations. Thus, taking the economical point of view for cerebral information processing might inspire theorists in efforts to construct more thermodynamically realistic models of neural and synaptic computations. These models would embrace relevant physics, rather than ignoring it, as advocated by William Bialek in a more general context of “biological physics” [102]. One such proposition, of a broad nature and generality, could be the principle of entropy maximization, which can be used to explain many types of data not only in neural systems but also in molecular biology [43,48,103]. However, its weakness is that it is based on equilibrium statistical mechanics, where time does not explicitly appear. Therefore, it is difficult to imagine (at least for the author) how this principle could be conceptually justified when applied to driven systems with stochastic dynamics, such as neurons and synapses in the non-stationary regime.

The examples described here were relatively simple, and they neglected some detailed features of real neurons and synapses. They were chosen because they can be treated analytically in a pedagogic way with explicit relationships between different quantities. Even for more complex models of neurons and synapses, the basic relationships between information and energy still hold, as described above; however, to reveal them requires heavy numerical calculations.

In the examples related to neural inference and synaptic plasticity, we used the idea of time-scale separation to derive analytical formulas. The dynamics of neurons and synapses can be quite complicated even for the relatively simple models we used because of the several time scales involved: from the neural firing rates time constants $\tau_{n0},\tau_{n}$ of the order of 1 s [19] to the synaptic plasticity time constants $\tau_{\theta }$ (∼10–20 s) and $\tau_{w}$ (∼100–600 s) [93,104]. Real brains have obviously much more intrinsic time scales, from milliseconds for some molecular processes (channels and receptors) [19], to seconds for neuromodulators, to hours or days for homeostatic processes [101], to months or years for developmental processes. This diversity of time scales is one of the main reasons for brain complexity, as many processes overlap and interact with one another [105,106,107]. In both of our examples, we observed that the stimulus variability, i.e., the external time scale, should be sufficiently slow to have any noticeable influence on neural and synaptic dynamics and on their information processing capability. Indeed, it seems that the slowness of the external stimulus can be a very important requirement for efficient computation not only in neural systems but generally in all biological systems with many interacting layers [88]. This is also the case for the efficiency of information propagation in the so-called critical regime of brain dynamics [108,109]. In this context, brain dynamics can be close to the critical point with long neural avalanches exhibiting power laws but only if the stimulus variability is slower than the duration of an avalanche [110].

In this perspective, the focus was on activities and information processing in individual neurons and synapses in small networks. Such an approach is similar in spirit to the physical approaches employed by others [12,17], where the authors analyzed energy constraints on the amount of learnt information. In these cases, the concepts of information and entropy production have a clear physical interpretation. However, in recent years, there are also other more global approaches, where the whole brain dynamics are analyzed from a thermodynamic point of view [15,111]. In such attempts, it is often difficult to interpret entropic quantities in terms of physical observables, because so many degrees of freedom, of different natures, are involved. In these global approaches, the goal seems to be different from the “physicality” of neurons and synapses. The authors rather focus on quantifying the irreversibility of global brain dynamics as described by the extent of a broken detailed balance on a level of whole macroscopic brain networks [15,16].

Despite many successes of computational and theoretical neuroscience (partly and briefly described in [112]), many traditional neurobiologists still neither understand it nor appreciate it. Even theoretical neuroscientists use models that often are not well grounded in neuronal reality, neglecting many physical aspects, e.g., energy, as irrelevant [19,20,23]. Theoretical neuroscience still needs a consistent and general theory to put diverse models and different theoretical pieces together in a unified way. I do hope that information thermodynamics, as developed in recent years by physicists, is a step in this ambitious direction. In this respect, the most promising approaches, in my opinion, would be the ones explicitly exploring simultaneously information and energy within stochastic thermodynamics by identifying the most important mechanisms on the micro- and mesoscopic levels, mainly in synapses, as they are important for learning and memory storing. Such approaches were initiated in [13,14,18]. However, to construct a general and powerful theory capable of making quantitative predictions requires much more, and it is not easy. The good starting point is the idea that the presence of nonpredictive information leads to energetic inefficiency [42]. Only retaining predicting (relevant) information in the memory makes sense from a thermodynamic point of view [113]. Making these ideas more concrete for “realistic” synapses could enhance our mechanistic understanding of synaptic plasticity in the context of acquiring and storing information.

## Appendix A

In this Appendix, we derive the population-averaged tuning curve in Equation (44). The summation in the expression $\bar{c}\left(v\right)=\left(1/N\right)\sum_{i=1}^{N}c_{i}\left(v\right)$ can be substituted by integration, i.e.,

(A1) $$\begin{array}{c} \bar{c}\left(v\right)\approx \int_{-\infty }^{\infty }\rho \left(u\right)c\left(v,u\right), \end{array}$$

where c(v,u) is the tuning curve for a given neuron as in Equation (42), i.e., $c\left(v,u\right)=r_{m}\exp\left[-{\left(v-u\right)}^{2}/\left(2\epsilon^{2}\right)\right]$, and ρ(u) is the distribution of neuronal preferences. We take ρ(u) in the form of the Gaussian such that

(A2) $$\begin{array}{c} \rho \left(u\right)=\frac{\exp\left(-\frac{u^{2}}{2\alpha^{2}}\right)}{\sqrt{2\pi \alpha^{2}}}, \end{array}$$

where α corresponds to the most likely range of stimulus velocities to which neurons respond. A straightforward calculation yields

(A3) $$\begin{array}{c} \bar{c}\left(v\right)=\frac{\epsilon r_{m}}{\sqrt{\alpha^{2}+\epsilon^{2}}}\exp\left[-\frac{v^{2}}{2(\alpha^{2}+\epsilon^{2})}\right]. \end{array}$$

This equation can be further approximated by noting that the most likely range of velocities α is much greater than the “window of selectivity” of a typical neuron ϵ, i.e., α/ϵ≫1. After this, we find Equation (44) in the main text.

## Appendix B

In this Appendix, we briefly show how to derive the steady-state mutual information $I{\left(\bar{r},v\right)}_{t\mapsto \infty }$ in Equation (52).

The mutual information in Equation (51), with exponentially decaying correlation for velocity of the stimulus, is proportional to the following integral *I*:

(A4) $$\begin{array}{c} I=\int_{0}^{t}dt_{1}\int_{0}^{t}dt_{2}\;e^{\left(t_{1}+t_{2}\right)/\tau_{n}}e^{-|t_{1}-t_{2}|/\tau_{c}}. \end{array}$$

We decompose the integral *I* into two integrals $I_{1}$ and $I_{2}$, such that $I=I_{1}+I_{2}$, where

(A5) $$\begin{array}{c} I_{1}=\int_{0}^{t}dt_{1}\int_{0}^{t_{1}}dt_{2}\;e^{\left(t_{1}+t_{2}\right)/\tau_{n}}e^{-\left(t_{1}-t_{2}\right)/\tau_{c}}, \end{array}$$

and

(A6) $$\begin{array}{c} I_{2}=\int_{0}^{t}dt_{1}\int_{t_{1}}^{t}dt_{2}\;e^{\left(t_{1}+t_{2}\right)/\tau_{n}}e^{-\left(t_{2}-t_{1}\right)/\tau_{c}}. \end{array}$$

A straightforward integration of $I_{1}$ and $I_{2}$ yields

(A7) $$\begin{array}{c} I=\frac{\tau_{n}^{2}\tau_{c}}{\left(\tau_{n}+\tau_{c}\right)}e^{2t/\tau_{n}}+\frac{2\tau_{n}^{2}\tau_{c}^{2}}{\left(\tau_{n}^{2}-\tau_{c}^{2}\right)}e^{t(\frac{1}{\tau_{n}}-\frac{1}{\tau_{c}})}+\frac{\tau_{n}^{2}\tau_{c}}{\left(\tau_{c}-\tau_{n}\right)}, \end{array}$$

after which we obtain Equation (52) in the main text.
